# P-Element-Induced Wimpy Testis (PIWI)-Interacting RNA-823/PIWIL1/DNMT3B/CDH1 as Potential Axis to Drive EMT, Stemness, and Tumor Aggressiveness in Ovarian Cancer Tissue Samples: An Integrative Computational and Clinical Insights

**DOI:** 10.3390/ijms27020823

**Published:** 2026-01-14

**Authors:** Fatma H. Shaker, Eman F. Sanad, Nader M. Ibrahim, Hesham Elghazaly, Shih-Min Hsia, Nadia M. Hamdy

**Affiliations:** 1 Department of Biochemistry and Molecular Biology, Faculty of Pharmacy, Ain Shams University, Cairo 11566, Egypt; 2 Department of Gynecology and Obstetrics, Faculty of Medicine, Ain Shams University, Cairo 11566, Egypt; 3 Department of Clinical Oncology, Faculty of Medicine, Ain Shams University, Cairo 11566, Egypt; 4 School of Nutrition and Health Sciences, College of Nutrition, Taipei Medical University, Taipei 110301, Taiwan; 5 School of Food Safety, Taipei Medical University, Taipei 110301, Taiwan; 6 Nutrition Research Center, Taipei Medical University Hospital, Taipei 110301, Taiwan; 7 Graduate Institute of Metabolism and Obesity Sciences, College of Nutrition, Taipei Medical University, Taipei 110301, Taiwan; 8 TMU Research Center for Digestive Medicine, Taipei Medical University, Taipei 110301, Taiwan

**Keywords:** ovarian cancer (OC), P-element-induced wimpy testis (PIWI)-interacting RNA-823 (piR-823), DNA methyltransferase 3B (DNMT3B), E-cadherin (CDH1), N-cadherin (CDH2), PIWIL1, bioinformatics, in silico analyses, epithelial–mesenchymal transition (EMT), NANOG, OCT4

## Abstract

Ovarian cancer (OC) remains the leading cause of death among gynecologic cancers. Most women diagnosed with OC at advanced stages eventually develop relapse and chemoresistance, leading to poor clinical outcomes. While piRNAs have emerged as critical regulators of gene expression and tumor biology, their specific roles in OC remain to be fully elucidated. This study integrated clinical and computational analyses to investigate the expression pattern and functional relevance of P-element-induced wimpy testis (PIWI)-interacting RNA-823 (piR-823) and its associated protein piwi-like RNA-mediated gene silencing 1 (PIWIL1)/DNA methyltransferase 3B (DNMT3B)/E-cadherin (CDH1) axis in OC tissues from 40 patients, with 20 non-cancer control samples. Expression profiling was performed using qPCR on OC and normal ovarian tissues, followed by correlation and regression analyses. Public databases, including GEPIA, TNM plot, and MethBank, were explored to validate gene expression, methylation status, and pathway enrichment. Our results revealed that piR-823, PIWIL1, and DNMT3B were significantly upregulated in OC tissues (*p* < 0.001, *p* = 0.009, and *p* < 0.001, respectively), and they correlated positively with each other and inversely with CDH1 expression. CDH2, OCT4, and NANOG were significantly upregulated (*p* = 0.011, *p* = 0.03, and *p* < 0.001, respectively), whereas CDH1 expression was significantly downregulated (*p* < 0.001) in OC tissues. In silico analyses supported DNMT3B-mediated CDH1 promoter methylation, epithelial–mesenchymal transition (EMT), and stemness pathway enrichment. Our integrated computational and clinical analyses indicate that the piR-823/PIWIL1/DNMT3B/CDH1 axis is a putative epigenetic regulator of EMT and cancer stemness in ovarian cancer. Additionally, piR-823 may serve as a promising prognostic biomarker and therapeutic target, offering novel insights into OC pathogenesis and treatment.

## 1. Introduction

Ovarian cancer (OC) is the most lethal gynecological malignancy, ranking eighth globally in both incidence and mortality, with only a 30–40% five-year overall survival rate [1]. Approximately 70% of OC patients are clinically diagnosed at advanced stages (stage III or IV) [2]. OC is a highly heterogeneous disease, comprising over 15 molecular and pathological subtypes, that complicates diagnosis and treatment decisions [3]. Despite standard treatments involving optimal cytoreductive surgery followed by chemotherapy, about 80% of patients experience drug-resistant recurrent OC [4]. The underlying mechanisms of OC chemoresistance and relapse are complex and multifactorial, attributed to epigenetic alterations [5,6], non-coding RNA (ncRNA), cancer stem cells (CSCs) [7], epithelial–mesenchymal transition (EMT) [8], as well as the tumor microenvironment [9]. The interplay between EMT and cancer stemness—mediated through genetic and epigenetic mechanisms—plays a central role in OC heterogeneity, aggressiveness, therapeutic resistance, and metastasis [10,11,12]. This interplay represents a promising area of research that drives the development of innovative prognostic markers and therapeutic strategies for chemo-resistant OC in the future.

P-element-induced wimpy testis (PIWI)-interacting RNAs (piRNAs) are a class of small ncRNAs ranging from 21 to 35 nucleotides. These RNAs, identified in germ and somatic cells, play critical roles in transposon silencing, epigenetic modification, and stem cell maintenance, thereby ensuring genome stability during germ-line development and spermatogenesis [13,14]. piRNAs specifically interact with a subfamily of Argonaute proteins, the PIWI proteins, to form the piRNA/PIWI complex, which regulates transposon silencing and epigenetic processes [15]. The PIWI protein family, comprising PIWIL1, PIWIL2, PIWIL3, and PIWIL4, is essential for piRNA biogenesis and piRNA-mediated gene regulation [16]. piRNAs exhibit tissue-specific expression patterns across various human tissues, and their aberrant expression is a hallmark of multiple tumor types, where they function as oncogenes or tumor suppressors [17]. However, the expression profiles and molecular mechanisms of piRNAs in OC remain largely unexplored.

piR-823 was first identified in gastric cancer and has since been detected in white blood cells, cancer cell lines, and blood plasma. Its involvement in malignancy has been reported in cancers such as breast cancer (BC) [18], colorectal cancer (CRC) [19,20], gastric cancer [21], esophageal cancer [22], renal cell carcinoma (RCC) [23,24], and multiple myeloma (MM) [25,26]. A key mechanism by which piR-823 contributes to cancer is through transcriptional and post-transcriptional gene silencing. At the transcriptional level, piR-823 can promote DNA methylation by recruiting DNA methyltransferases (DNMTs), as observed in BC [18] and MM [25]. Specifically, DNA methylation mediated by DNA methyltransferase 3B (DNMT3B) plays a crucial role in regulating gene expression and is strongly associated with tumor initiation and progression across various tumors [27]. High DNMT3B expression is associated with silencing of tumor suppressor genes, such as E-cadherin (CDH1) [28,29], the promotion of EMT [30], the maintenance of cancer stemness [18], and chemoresistance. This epigenetic mechanism has also been widely implicated in the progression of OC [30,31].

In this study, we investigated the clinical and molecular significance of piR-823 in OC and explored its potential regulatory role in tumor progression through a proposed epigenetic axis involving PIWIL1, DNMT3B, and CDH1. By integrating experimental expression profiling and computational bioinformatics analyses, we characterized the epigenetic and regulatory mechanisms by which piR-823 contributes to EMT, cancer stemness, and tumor aggressiveness, and evaluated the potential of piR-823 as a prognostic biomarker and therapeutic target in OC. These objectives were examined in tissue samples from patients and controls.

## 2. Results

### 2.1. Demographic, Clinical, and Pathological Characteristics of Study Participants

**Figure 1:**
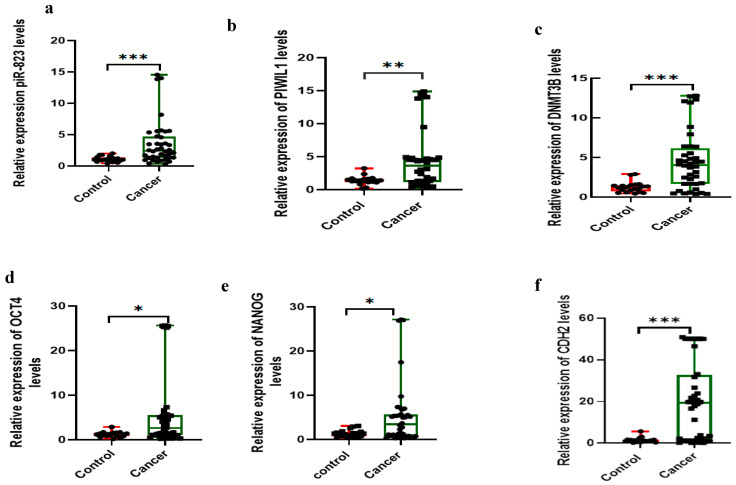

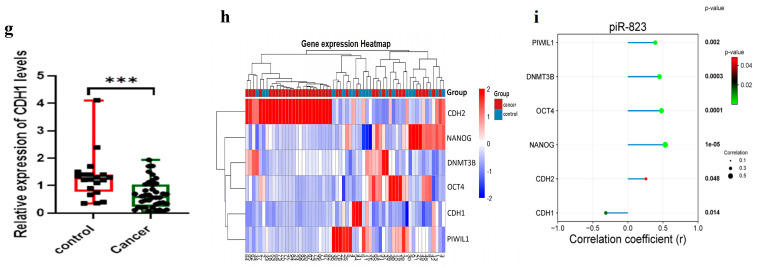
Expression levels of piR-823 and the investigated mRNAs in the study participants (n = 60). (**a**) piR-823, (**b**) PIWIL1, (**c**) DNMT3B, (**d**) OCT4, (**e**) NANOG, (**f**) CDH2, (**g**) CDH1. Statistics were computed using Mann–Whitney tests. * Statistical significance *p*-value < 0.05. ** Statistical significance is at *p*-value < 0.01. *** Statistical significance is at *p*-value < 0.001. (**h**) Heatmap of gene expression in OC and control samples. Each gene’s expression was standardized using Z-score normalization across samples (rows = genes, columns = individual samples). (**i**) Spearman correlation dot line plot of piR-823 with PIWIL1, DNMT3B, OCT4, NANOG, CDH2, and CDH1 drawn using SR plot (https://www.bioinformatics.com.cn/srplot) (accessed on 7 January 2024) where the green dot denotes *p*-values closer to 0.02 and the red one denotes *p*-values closer to 0.04, indicating weaker significance. The size of each symbol denotes magnitude of the correlation coefficient (*r*).

The study comprised 40 OC patients and 20 female controls, as shown in Table 1. The mean age (years) ± standard error of the mean (S.E.M.) of OC patients and controls was 53.55 ± 1.77 and 53.35 ± 2.03, respectively, with no significant difference between the groups. Similarly, premenopausal status was observed in 19 OC cases (57.1%) and 8 controls (68%), showing no significant difference. Regarding gene expression, piR-823, PIWIL1, DNMT3B, OCT4, NANOG, and CDH2 were significantly upregulated in OC patients compared to the controls (*p* < 0.001, *p* = 0.009, *p* < 0.001, *p* = 0.011, *p* = 0.03, and *p* < 0.001, respectively) as illustrated in Figure 1a–f, and detailed in Table 1. In contrast, CDH1 expression was significantly downregulated in OC patients compared to controls (*p* < 0.001), as shown in Figure 1g and in Table 1. To visualize these findings, a heatmap (Figure 1h) was constructed using the R package R-4.3.3 (pheatmap) from the processed qRT-PCR dataset, focusing on DNMT3B, OCT4, NANOG, CDH2, CDH1, and PIWIL1. The heatmap revealed that PIWIL1, DNMT3B, OCT4, NANOG, and CDH2 were increased in most ovarian cancer samples, while CDH1 was downregulated, thus delineating clear expression differences between cancer and control samples.

### 2.2. piR-823 Expression Pattern in OC (n = 40) Stratified Subgroups According to FIGO Stages, Grades, LNM, and Ascites

**Figure 2:**
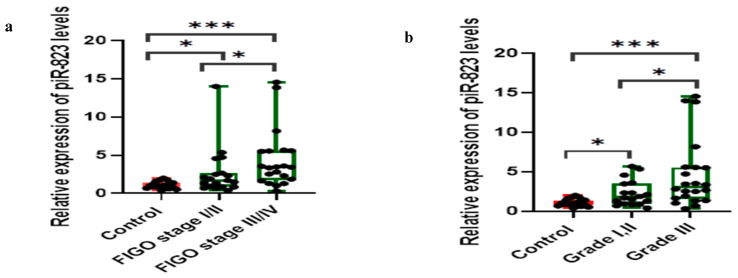

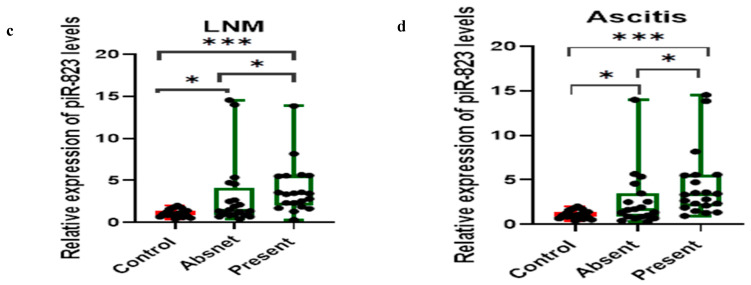
piR-823 expression pattern in OC patients (n = 40) stratified subgroups: (**a**) FIGO stages I/II (n = 19) vs. stages III/IV (n = 21), (**b**) tumor grades I, II (n = 18) vs. grade III (n = 22), (**c**) LNM absent (n = 20) vs. present (n = 20), (**d**) ascites absent (n = 19) vs. present (n = 21). Statistics were computed using Kruskal–Wallis tests. * Statistical significance *p*-value < 0.05. *** Statistical significance is at *p*-value < 0.001. [LNM, lymph node metastasis].

The expression levels of piR-823 were significantly elevated in patients with advanced FIGO stages (III and IV) (n = 21) and higher histological grades (Grade III) (n = 22) compared to those with early-stage disease (FIGO stages I and II) (n = 19) and lower histological grades (Grades I and II) (n = 18) (*p* = 0.027 and 0.022, respectively), and when compared to the control group (Figure 2a,b). Also, OC patients exhibiting positive LNM (n = 20) and ascites (n = 21) demonstrated markedly higher piR-823 expression levels relative to those without LNM (n = 20) and ascites (n = 19) (*p* = 0.02 for both comparisons) and the control group (Figure 2c,d). Collectively, these findings suggest that elevated piR-823 expression is associated with unfavorable oncological outcomes, indicating its potential role as a biomarker for disease progression and severity in OC.

### 2.3. Relations Between the Investigated Molecular Biomarkers and Patient Clinicopathological Factors

The expression levels of piR-823, PIWIL1, DNMT3B, OCT4, NANOG, CDH2, and CDH1 across OC stratified groups are summarized in Table 2. When stratifying gene expression by clinicopathological features, piR-823 was significantly upregulated in patients with advanced FIGO stage (III–IV) (*p* = 0.027), high histological grade (GIII) (*p* = 0.022), positive LNM (*p* = 0.02), and presence of ascites (*p* = 0.02). Furthermore, expression of OCT4 was significantly elevated in patients with high-grade tumors (*p* = 0.012) and in those with ascites (*p* = 0.023). Also, NANOG demonstrated higher expression in ascitic cases (*p* = 0.02). No significant associations were found for other clinicopathological variables (age, tumor size, menopausal status, tumor type, or distant metastasis). These findings suggest that increased expression of piR-823, NANOG, and OCT4 is associated with more aggressive and advanced tumor features, highlighting their potential role in OC progression.

Moreover, the correlations between the investigated molecular biomarkers and clinicopathological characteristics were detailed in Table 3. Notably, Spearman correlation analysis revealed that OCT4 expression was significantly associated with tumor site (*r* = 0.432, *p* = 0.005), showing higher expression in bilateral ovarian tumors. Furthermore, piR-823 expression was positively correlated with tumor grade (*r* = 0.348, *p* = 0.028), suggesting elevated levels in poorly differentiated tumors. NANOG expression negatively correlated with tumor size (*r* = −0.383, *p* = 0.015). No significant correlations were observed between gene expression and age, menopausal status, tumor type, FIGO stage, LNM or distant metastases, or presence of ascites for the other genes assessed.

Spearman correlation analysis between the expression levels of piR-823, PIWIL1, DNMT3B, OCT4, NANOG, CDH2, and CDH1 in OC patients (n = 40) and controls (n = 20) revealed several significant associations (Figure 1i and Table 4). Notably, piR-823 expression exhibited strong positive correlations with PIWIL1 (*r* = 0.391, *p* = 0.002), DNMT3B (*r* = 0.451, *p* < 0.001), OCT4 (*r* = 0.479, *p* < 0.001), and NANOG (*r* = 0.533, *p* < 0.001). Additionally, piR-823 was positively correlated with CDH2 (*r* = 0.257, *p* = 0.048) and negatively correlated with CDH1 (*r* = −0.315, *p* = 0.014), suggesting its potential involvement in EMT. DNMT3B showed strong positive correlation with PIWIL1 (*r* = 0.35, *p* = 0.006), OCT4 (*r* = 0.45, *p* < 0.001), NANOG (*r* = 0.389, *p* = 0.002), and CDH2 (*r* = 0.517, *p* < 0.001) while negatively correlated with CDH1 (*r* = −0.308, *p* = 0.017) as shown in Table 4. These findings were further supported by data from the Organoid database, which revealed positive correlations between DNMT3B and both NANOG and CDH2, and a negative correlation with CDH1 across OC datasets (Supplementary Figure S1). Similarly, PIWIL1 showed strong positive correlation with piR-823, DNMT3B, OCT4 (*r* = 0.436, *p* < 0.001), and NANOG (*r* = 0.42, *p* < 0.001), suggesting coordinated upregulation of these markers.

Further correlation analysis between the investigated molecular biomarkers and OC patients’ subgroups was performed in Table 5. In the entire OC group, piR-823 expression showed significant positive correlations with PIWIL1 (*r* = 0.402, *p* = 0.013), OCT4 (*r* = 0.421, *p* = 0.007), and NANOG (*r* = 0.405, *p* = 0.01). Subgroup analysis revealed stronger correlations in specific clinical contexts. In patients aged ≥ 50 years, piR-823 expression was significantly correlated with PIWIL1 (*r* = 0.514, *p* = 0.004), OCT4 (*r* = 0.400, *p* = 0.032), and NANOG (*r* = 0.490, *p* = 0.007). The serous subtype demonstrated a particularly strong correlation between piR-823 and OCT4 (*r* = 0.664, *p* = 0.007) and a moderate correlation with CDH2 (*r* = 0.547, *p* = 0.035). Similarly, in patients with bilateral tumors, piR-823 expression correlated significantly with OCT4 (*r* = 0.602, *p* = 0.008) and CDH2 (*r* = 0.508, *p* = 0.031). Among patients with advanced FIGO stage (III/IV), a notable correlation was observed between piR-823 and PIWIL1 (*r* = 0.595, *p* = 0.004). In those with positive ascites, piR-823 remained significantly associated with PIWIL1 (*r* = 0.482, *p* = 0.027). In those with tumor size > 10 cm, piR-823 exhibited a highly significant correlation with NANOG (*r* = 0.451, *p* = 0.014). Remarkably, in postmenopausal patients, piR-823 exhibited a significant correlation with NANOG (*r* = 0.733, *p* < 0.001). The subgroup-specific analysis of piR-823 correlations provides deeper insights into its interactions with related biomarkers in driving OC progression. The consistent positive association between piR-823 and PIWIL1, especially in older, advanced-stage, and ascites-positive patients, reinforces the hypothesis that PIWIL1 plays a pivotal role in piR-823 biogenesis and function, particularly in more aggressive or advanced diseases. The strong correlations of piR-823 with OCT4 and NANOG across multiple subgroups, particularly in serous subtypes, bilateral tumors, and postmenopausal patients, highlight a possible link between piR-823 expression and stemness-associated transcription factors. Additionally, the correlation of piR-823 with mesenchymal marker CDH2 in serous and bilateral tumors suggests a role in enhancing EMT.

### 2.4. In-Silico Search and Bioinformatics Analysis of the Investigated Markers

**Figure 3:**
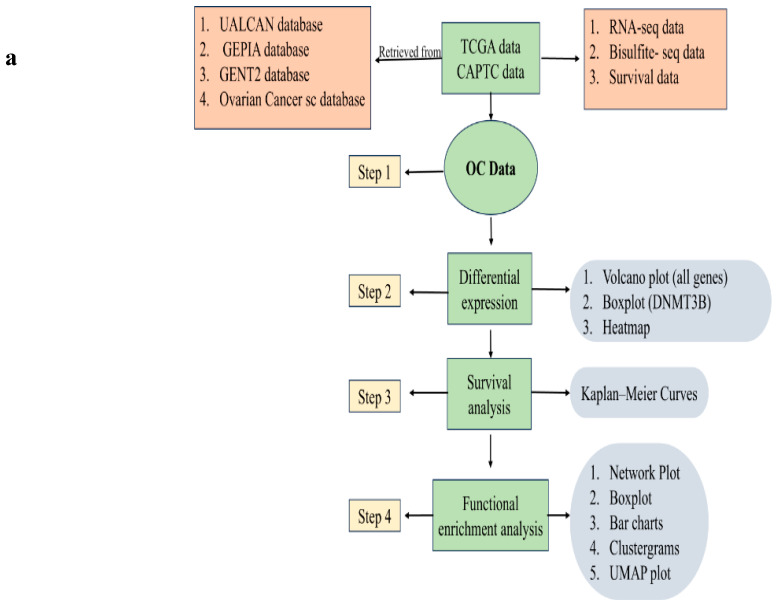

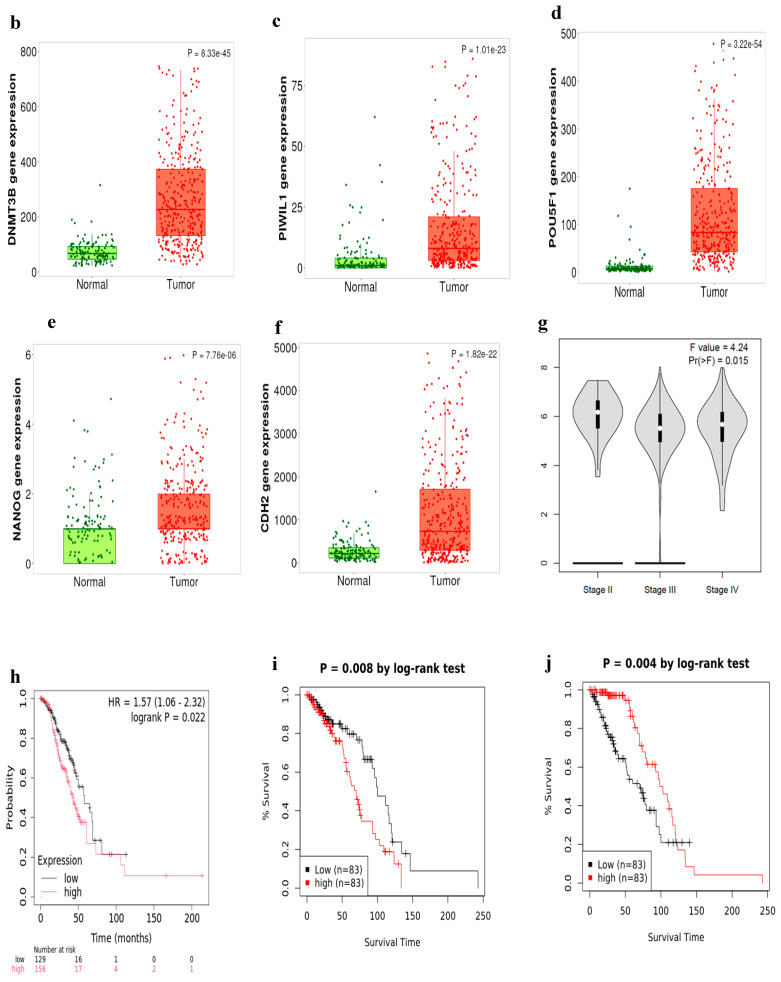
In-silico search and bioinformatics analysis of the investigated markers using OC datasets. (**a**) Data processing flowchart. Boxplot presents the expression of (**b**) DNMT3B, (**c**) PIWIL1, (**d**) OCT4 (POU5F1), (**e**) NANOG, and (**f**) CDH2 in OC tissues (red) and normal tissues (green) using TNM plot database (https://tnmplot.com/analysis/) (accessed on 12 October 2025). (**g**) Violin plot illustrating CDH1 gene expression across OC stages II, III, and IV using GEPIA database. Kaplan–Meier survival curves comparing overall survival between patients with low (black line) and high (red line) expression of following genes (**h**) DNMT3B, (**i**) CDH2, (**j**) CDH1.

In this study, Figure 3a outlines the data analysis workflow. To compare marker expression in OC and normal tissues, we analyzed RNA-seq data for DNMT3B, PIWIL1, NANOG, OCT4 (POU5F1), and CDH2 using the TNM plot database. As shown in Figure 3b–f, expression of DNMT3B, PIWIL1, NANOG, OCT4, and CDH2 was significantly elevated in tumor samples compared to normal controls (*p* = 8.33 × 10^−45^, 1.01 × 10^−23^, 7.76 × 10^−6^, 3.22 × 10^−54^, and 1.82 × 10^−22^, respectively), indicating strong tumor-specific upregulation of these genes. Further analysis of CDH1 expression across different OC tumor stages revealed a progressive decrease from Stage II to Stage IV (F = 4.24, *p* = 0.015), as shown in Figure 3g. To assess the clinical relevance of these genes, Kaplan–Meier survival analyses were performed. The results indicated that patients with lower DNMT3B expression exhibited a significantly improved overall survival (OS) probability compared to those with higher DNMT3B levels (*p* = 0.022) (Figure 3h). Similarly, patients with elevated CDH2 expression exhibited significantly reduced OS probabilities *(p* = 0.008) (Figure 3i). However, OC patients with higher CDH1 expression show significantly longer overall survival (*p* = 0.004), indicating that CDH1 acts as a protective prognostic marker (Figure 3j).

### 2.5. Association Between piR-823, DNMT3B, and Cancer Stemness in OC

**Figure 4:**
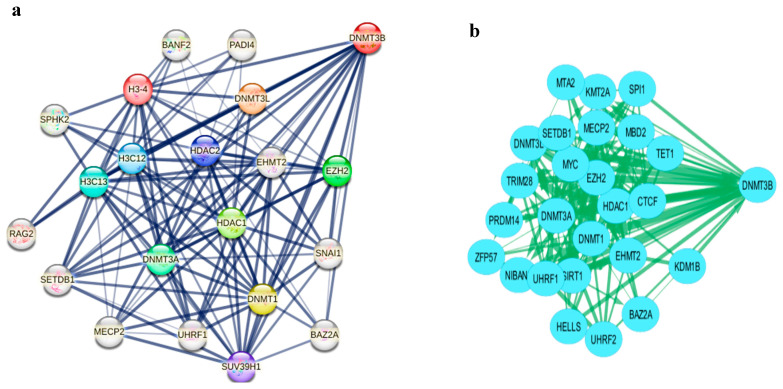

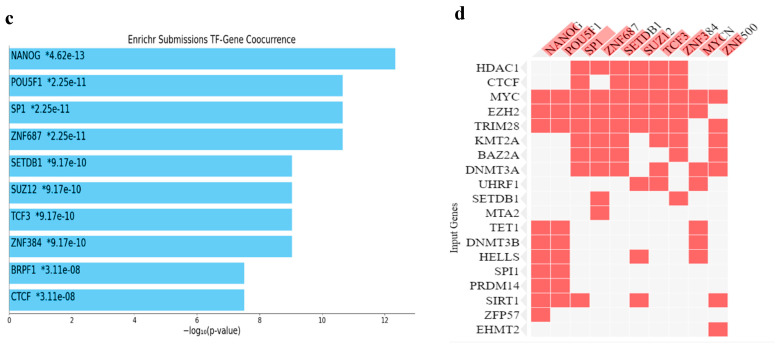
Association between piR-823, DNMT3B, and cancer stemness in OC: (**a**) PPI network of DNMT3B interactions via STRING (https://string-db.org/cgi/network?taskId=bgqR2f3LeTuD&sessionId=bBgfMOILrJaR) (accessed on 13 January 2024), (**b**) PPI network DNMT3B using INDRA database (https://www.ndexbio.org/viewer/networks/353ff272-84ab11ecb3be0ac135e8bacf?query=DNMT3B+&queryType=firstStepNeighborhood&maximizeResultView=true) (accessed on 15 January 2024), (**c**) Cluster gram for top enriched genes of DNMT3B using Enrichr database (https://maayanlab.cloud/Enrichr/enrich) (accessed on 22 January 2024), * denotes the statistical significance values (*p*-values), and (**d**) Bar chart of top enriched genes of DNMT3B using Enrichr database (https://maayanlab.cloud/Enrichr/enrich) (accessed on 22 January 2024).

To investigate the functional role of DNMT3B, STRING database analysis was conducted, generating a PPI network with 21 nodes (genes) and 119 edges (protein interactions) (enrichment *p* < 1.0 × 10^−16^) (Figure 4a). The results indicated that regulation of stem cell maintenance and differentiation were among the most significant GO biological processes associated with DNMT3B. Complementary analyses using the STITCH database, which integrates protein-chemical interaction data, confirmed that DNMT3B is strongly linked to the regulation of stem cell maintenance pathways based on ovarian RNA-seq data from the Human Protein Atlas (Supplementary Figure S3). An additional mechanistic network was derived from the INDRA database, revealing key protein interactions involving DNMT3B (Figure 4b), followed by functional enrichment analysis using the Enrichr database, which supported these findings, highlighting the involvement of NANOG and OCT4 -key regulators of cancer stemness- as top overlapping genes with DNMT3B (Figure 4c,d). The KEGG pathway map of the signaling pathway regulating stem cell pluripotency (Supplementary Figure S4) highlights the roles of NANOG and OCT4 as core effectors in stem cell regulation. Together with the correlations shown in Table 4 and Table 5, which demonstrated significant associations between piR-823, DNMT3B, OCT4, and NANOG, suggest that piR-823 and DNMT3B may cooperatively contribute to cancer stemness in OC.

### 2.6. The Association of piR-823, DNMT3B, and Epithelial–Mesenchymal Transition (EMT) in OC

**Figure 5:**
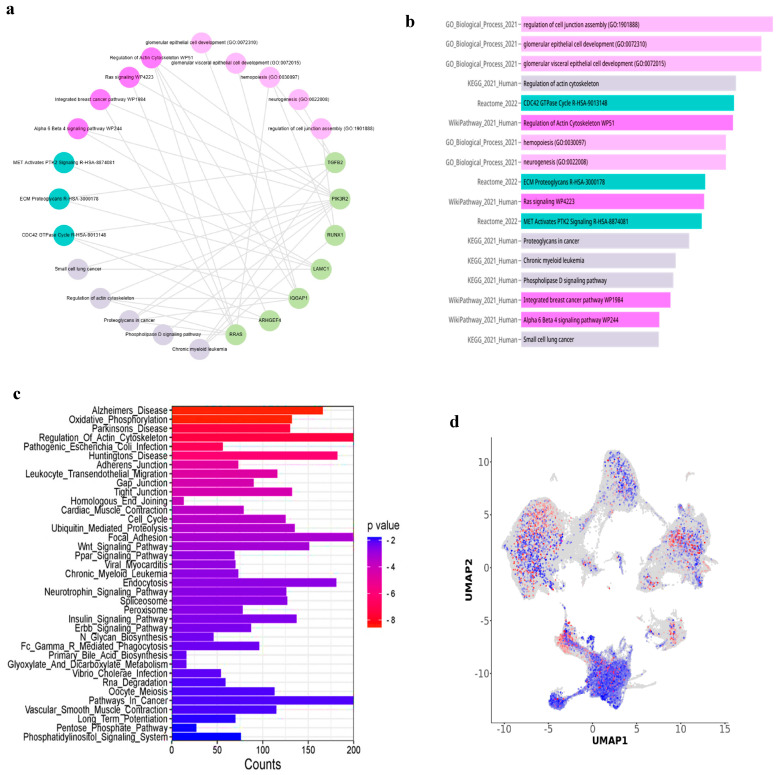

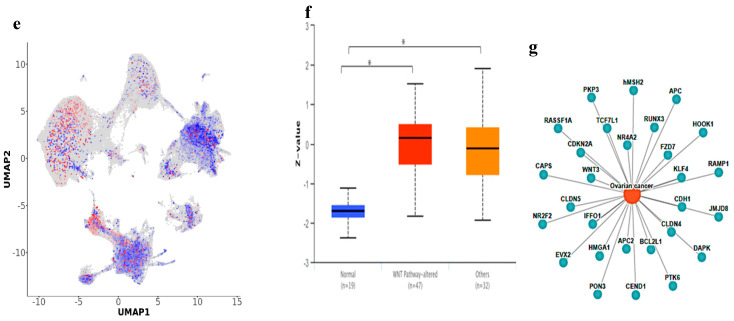
The association of piR-823, DNMT3B, and epithelial–mesenchymal transition (EMT) in OC: (**a**) Network-based pathway enrichment visualization showing functionally grouped terms enriched among the 107 predicted piR-823 target genes (identified using the sRNAtool database) (accessed on 20 October 2025) (**b**) Bar plot of top enriched biological processes and pathways using Enricher-KG (accessed on 20 October 2025), (**c**) enriched horizontal bars of the targets in KEGG Pathways Enrichment Analysis of DNMT3 targets using ENCOR database (https://rnasysu.com/encori/rriPathways.php?source=mRNA&flag=RNA&clade=mammal&genome=human&assembly=hg38&RNA=DNMT3B&interNum=&expNum=&pathway=) (accessed on 20 January 2024), (**d**) UMAP plot of CDH1 (blue dots) and DNMT3B in Ovarian samples (red dots) (accessed on 20 May 2025), (**e**) UMAP plot of CDH2 (blue dots) and DNMT3B (red dots) in ovarian samples (accessed on 20 May 2025), (**f**) Expression of DNNT3B in ovarian CAPTC dataset related to Wnt pathway signaling (https://ualcan.path.uab.edu/cgi-bin/CPTAC-Result-Phos.pl?genenam=DNMT3B&ctype=OV) (accessed on 16 September 2024), * significant difference between groups at *p* = 0.05. (**g**) network showing DNA-methylated genes in OC using MethBank database (https://ngdc.cncb.ac.cn/methbank/curation) (accessed on 16 September 2024).

To investigate the potential biological roles of piR-823 in OC, we retrieved 107 predicted target genes using the sRNAtool database (Table S1) and performed enrichment analysis using multiple gene set libraries in Enricher-KG (Figure 5a,b). In the network-based pathway enrichment analysis (Figure 5a), the top-enriched pathways and biological processes were grouped into distinct functional clusters, including “glomerular epithelial cell development” and “regulation of cell junction assembly”, suggesting a potential role in epithelial differentiation and polarity. Another cluster was enriched in cell adhesion and signaling pathways, such as “regulation of actin cytoskeleton” and “Ras signaling.” These results suggest that piR-823 may modulate genes involved in epithelial integrity, cytoskeletal remodeling, and oncogenic signaling, all of which are crucial in cancer cell invasion and metastasis. To further elucidate the functional pathways associated with DNMT3B, KEGG pathway enrichment analysis was performed using the StarBase database. The analysis identified pathways related to Actin Cytoskeleton Regulation, Adherens Junctions, Gap Junctions, and Tight Junctions among the top 10 enriched pathways (Figure 5c). Moreover, a heatmap derived from the GSEA database for the KEGG Adherens Junction pathway (Supplementary Figure S5) illustrates the expression of 73 genes involved in this pathway, including downregulated CDH1 and upregulated CDH2 across OC cell lines. The correlation plot in (Supplementary Figure S2) indicates that upregulation of CDH2 is associated with a mesenchymal shift, and downregulation of CDH1 correlates with loss of epithelial characteristics in OC. Based on the UMAP plot retrieved from the Ovary Cancer sc Database (Figure 5d), the plot suggests an inverse spatial expression pattern between CDH1 and DNMT3B in several regions. However, more extensive overlapping regions were observed (Figure 5e), suggesting a co-expression of CDH2 and DNMT3B, possibly reflecting a positive correlation. Notably, the Wnt signaling pathway, a well-established regulator of the Adherens junctions (Supplementary Figure S6), emerged as a significant pathway associated with DNMT3B interactions (Figure 5c). CPTAC data showed significantly higher DNMT3B expression in Wnt-altered OC samples compared to controls (*p* = 3.4 × 10^−9^) (Figure 5f). Additionally, functional enrichment analysis using the STRING database identified positive regulation of EMT as one of the top GO biological processes (enrichment strength = 1.7, *p* = 0.004). To explore epigenetic regulation, MethBank database analysis revealed a network of differentially methylated genes in OC. Specifically, the CDH1 gene promoters were found to be hypermethylated in OC tissues compared to controls (Figure 5g). Given the strong correlation of piR-823 with DNMT3B and EMT markers (CDH1, CDH2) as well as its association with LNM, this suggests the potential involvement of piR-823 and DNMT3B in EMT in OC.

### 2.7. Multiple Linear Regression Analysis Model

To assess how piR-823 and DNMT3B influence the expression of stemness markers and EMT-related markers, both simple and multiple linear regression analyses were conducted in Table 6 and Table 7. Since the data were non-parametric, they were transformed into log scale prior to analysis. Simple linear regression analyses shown in Table 6, where independent variables are either piR-823 or DNMT3B and dependent variables include OCT4, NANOG, CDH2, and CDH1, revealed a significant positive association between piR-823 and DNMT3B expression (R^2^ = 0.158, B = 0.46, *p* = 0.002). OCT4 and NANOG showed even stronger associations with piR-823 (OCT4: R^2^ = 0.21, B = 0.63, *p* < 0.001; NANOG: R^2^ = 0.258, B = 0.69, *p* < 0.001). Notably, CDH1 exhibited a significant negative correlation with piR-823 (*B* = −0.31, *p* = 0.018), suggesting a possible inverse regulatory relationship.

As shown in Table 7, multiple linear regression analysis, with OCT4, NANOG, CDH2, and CDH1 as dependent variables, and piR-823 and DNMT3B as independent variables, revealed that both piR-823 and DNMT3B significantly predicted the expression of stemness-related genes OCT4 and NANOG, with piR-823 showing a stronger influence (OCT4: *B* = 0.49, *p* = 0.005; NANOG: *B* = 0.56, *p* = 0.001). In contrast, EMT marker CDH2 expression was significantly associated with DNMT3B (*B* = 0.86, *p* = 0.001), but not with piR-823. CDH1 expression was not significantly predicted by either variable. These findings suggest a functional role for piR-823 in regulating pluripotency-related pathways, while DNMT3B appears to impact both stemness and EMT-related gene expression.

#### 2.7.1. Stemness Markers Analysis

**Figure 6:**
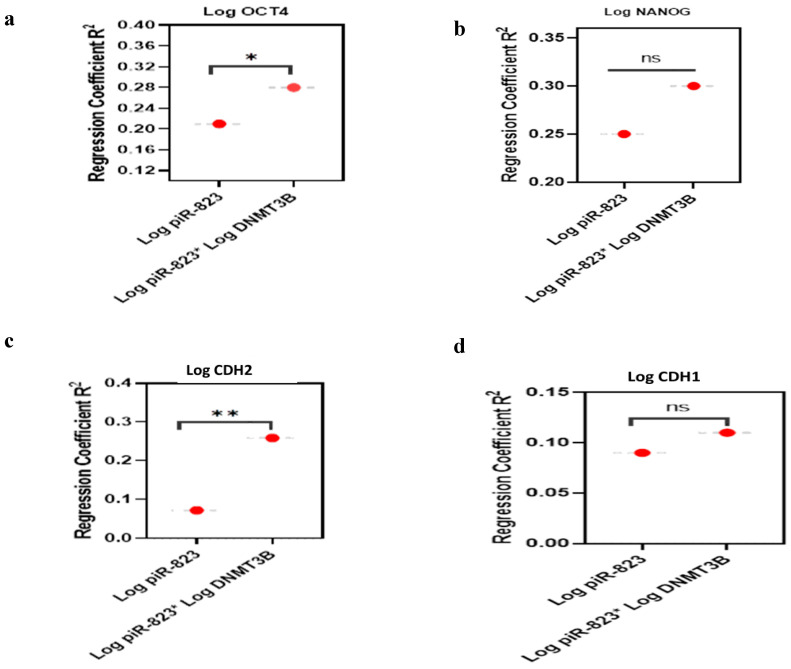
Multiple Linear Regression Analysis Model: (**a**) OCT4, (**b**) NANOG, (**c**) CDH2, and (**d**) CDH1. The red dot is the position for the estimate of each regression coefficient R^2^ for piR-823 and DNMT3B against. * Significant statistical difference *p* < 0.05. ** Significant statistical difference *p* < 0.01.

For the OCT4 model, piR-823 alone accounted for 21% (R^2^ = 0.21) of the variation in OCT4 expression. When DNMT3B was included as a second predictor, the model’s explanatory power increased to 28% (R^2^ = 0.28), with an R^2^ change of 0.06, which was statistically significant (*p* = 0.033). The overall model was significant (*p* = 0.003) (Figure 6a). This suggests that piR-823 and DNMT3B collectively explain 28% of the variation in OCT4 expression. For NANOG, piR-823 alone explained 25.8% (R^2^ = 0.258) of its variation. When DNMT3B was added, the model’s explanatory power increased to 30.7% (R^2^ = 0.307), with an R^2^ change of 0.187, which was statistically significant (*p* = 0.001). The overall model was highly significant (*p* < 0.0001) (Figure 6b). This indicates that piR-823 and DNMT3B positively influence 30.7% of NANOG expression variability.

#### 2.7.2. EMT-Related Markers Analysis

For CDH2, piR-823 alone accounted for 7.8% (R^2^ = 0.078) of its variation. With DNMT3B as a second predictor, the model’s explanatory power rose to 25.9% (R^2^ = 0.259), with an R^2^ change of 0.049, which was non-significant (*p* = 0.51). However, the overall model was significant (*p* < 0.001) (Figure 6c). This suggests that piR-823 and DNMT3B collectively explain 26% of the variation in CDH2 expression. Conversely, for CDH1, piR-823 alone explained 9.3% (R^2^ = 0.093) of its variation. Adding DNMT3B increased the explanatory power to 11% (R^2^ = 0.11), with an R^2^ change of 0.024, which was not statistically significant (*p* = 0.21) (Figure 6d). Despite this, the overall model remained significant (*p* = 0.028) Notably, piR-823 and DNMT3B showed a negative association, collectively explaining 11% of the variability in CDH1 expression (*p* = 0.028).

Both simple and multiple linear regression analyses indicated that piR-823 and DNMT3B have a positive regulatory effect on stemness markers (OCT4 and NANOG), while DNMT3B mainly governed EMT-related marker (CDH2) expression, further supporting their involvement in tumor progression and metastasis.

### 2.8. Diagnostic and Prognostic Utility of piR-823 in OC Using the ROC Curve

**Figure 7:**
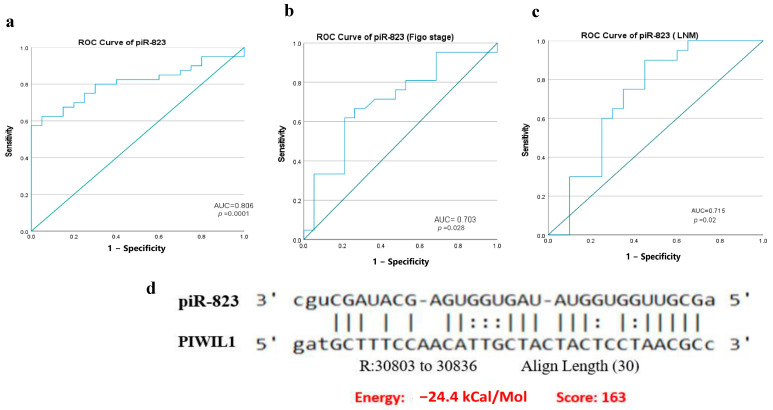
Diagnostic and prognostic utility of piR-823 and its interaction with PIWIL1. (**a**) ROC curve illustrating the expression level of piR-823 was able to distinguish OC tissues from normal tissues, with AUC value of 0.0.806 (*p* = 0.0001). (**b**) ROC curve illustrating the expression level of piR-823 was markedly associated with higher FIGO stage, with AUC of 0.7 (*p* = 0.028). (**c**) ROC curve illustrating the expression level of piR-823 was markedly associated with LNM, with AUC of 0.715 (*p* = 0.02). (**d**) Predicted multiple sequence alignments of piR-823 with PIWIL1 using the miRanda algorithm (accessed on 16 September 2024) [AUC, the area under the curve; ROC, receiver operating characteristic; LNM, lymph node metastasis.].

We further evaluated the diagnostic potential of piR-823 using ROC curve analysis. The results demonstrated that piR-823 tissue levels were a valuable biomarker for distinguishing OC tissue from normal controls, with an AUC of 0.806 (95% CI: [0.698–0.914], *p* = 0.0001) (Figure 7a). By applying Youden’s J statistic to determine the optimal cutoff, piR-823 showed a sensitivity of 72.5% and specificity of 75%, effectively distinguishing OC patients from normal controls. To further assess the prognostic significance of piR-823 in OC, ROC curve analysis was performed to differentiate between advanced and early FIGO stages, as well as LNM-positive and LNM-negative cases. The results revealed significantly higher piR-823 levels in OC tissues with advanced FIGO stage compared to early-stage cases, with an AUC of 0.7 (95% CI: [0.538–0.868], *p* = 0.028) (Figure 7b). Similarly, piR-823 expression was markedly elevated in LNM-positive cases compared to LNM-negative cases, with an AUC of 0.715 (95% CI: [0.549–0.881], *p* = 0.02) (Figure 7c). These findings highlight the potential of piR-823 as both a diagnostic and prognostic biomarker for OC.

### 2.9. Correlation and Potential Interaction Between piR-823 and PIWIL1 in OC

Expression levels of piR-823 were significantly correlated with PIWIL1 expression levels in OC patients as well as in different OC stratified groups, as shown in Table 5, such as patients of Age ≥ 50, patients of higher FIGO stage, and patients with positive ascites, implying the involvement of PIWIL1 in piR-823 biogenesis. Further, to determine whether piR-823 had a putative binding site within PIWIL1 mRNA, the miRanda algorithm was used to predict target interaction between piR-823 and PIWIL1. The results suggested that the entire length of piR-823 can participate in complementary base pairing with PIWIL1 (Figure 7d), which suggests the potential involvement of PIWIL1 in piR-823 molecular functions by forming PIWIL/piRNA complex (PRISC).

## 3. Discussion

Ovarian cancer (OC) is a highly heterogeneous disease driven by complex epigenetic alterations that affect numerous oncogenes and tumor suppressor genes. These alterations disrupt critical signaling pathways involved in DNA repair, cell proliferation, apoptosis, cell adhesion, and cell motility. Its distinct patterns of progression and recurrence suggest a hierarchical structure supported by a tumor microenvironment that sustains cancer stemness and promotes EMT. This process enhances the invasive and metastatic potential of cancer [32,33,34]. Consequently, an in-depth investigation into the key molecular mechanisms underlying OC pathogenesis and progression is both necessary and holds promise for identifying novel therapeutic targets. piRNAs have emerged as critical regulators of transcription and post-transcriptional processes in cancer biology. Recent studies reported dysregulated piRNAs in OC with potential clinical relevance. For example, piR-1919609 is frequently overexpressed in primary and recurrent OC, correlating with poor prognosis and platinum resistance [1]. Conversely, piR-28846 is downregulated in OC, suppresses tumor growth in vitro, and exhibits therapeutic potential in xenograft and organoid models [35]. Although emerging data suggest that piRNAs are involved in OC progression, the field remains relatively nascent. Previous studies have demonstrated that piR-823 exhibits differential expression patterns and functions across various cancer types. In most cancers, piR-823 is upregulated, including in BC [36], CRC [37,38], liver cancer [39,40], and MM [26]. Conversely, piR-823 is downregulated in gastric cancer [41] and renal cancer [42]. In the current study, we characterized the expression of piR-823 in OC for the first time. Our findings revealed a significant upregulation of piR-823 in OC tissues compared to normal tissues.

Moreover, piRNAs have shown promising potential as prognostic biomarkers in cancer. Several studies have demonstrated associations between piR-823 expression and tumor-node-metastasis (TNM) stage, histological type, cancer progression, and overall survival in various cancer types [43,44]. Our findings revealed that piR-823 overexpression was significantly associated with LNM, advanced FIGO staging, ascites, and high histological grade, further supporting its potential prognostic value in OC. piRNAs are promising candidates for cancer diagnostics [45]. Serum levels of piR-823 are considered a diagnostic biomarker in several cancers [23,38,41]. Furthermore, tissue levels of piR-823 have demonstrated utility as biomarkers for distinguishing cancerous from normal tissues in ESCC [22] and RCC [24]. Consistently, our findings revealed a high AUC value of 0.806, highlighting the diagnostic potential of piR-823 in OC detection.

piRNAs have been implicated in cancer initiation and progression by regulating gene expression at both transcriptional and post-transcriptional levels [46,47]. At the transcriptional level, piRNAs mediate epigenetic modifications, including aberrant DNA methylation and histone modification deposition [48]. Crosstalk between piRNAs and epigenetic elements involving DNMTs profoundly affects genomic stability and gene expression. This interaction may dysregulate key signaling pathways, thereby promoting tumorigenesis [49,50]. In MM, piR-823 has been shown to promote DNA methylation through DNMT3A and DNMT3B, enhancing the tumorigenic capacity of MM cells by suppressing the tumor suppressor gene p16INK4A [25]. Additionally, piR-823, activated by myeloid-derived suppressor cells, enhances the stem-like properties of MM cells by interacting with DNMT3B [51]. Similarly, piR-823 exhibits oncogenic activity in esophageal squamous cell carcinoma (ESCC) by activating aberrant DNA methylation through DNMT3B [22]. In BC, piR-823 upregulated DNMTs expression levels, promoted DNA methylation of the adenomatous polyposis coli (APC) gene, activated Wnt signaling, and induced cancer cell stemness in luminal subtype cells [18]. Consistent with these studies, our correlation analysis (Table 4) showed a strong positive correlation between piR-823 and DNMT3B expression, and simple linear regression analysis (Table 6) revealed that piR-823 significantly influences the variation in DNMT3B expression (R^2^ = 0.158, *p* = 0.002, CI: [0.18–0.73]). These findings suggest that piR-823 may exert oncogenic activity in OC by activating aberrant DNA methylation via DNMT3B; however, further validation is required.

From a translational perspective, piR-823 and DNMT3B may represent a promising therapeutic target. Given the emerging interest in targeting oncogenic ncRNAs, therapeutic inhibition of piR-823 using small RNA-based approaches may offer a novel strategy to suppress cancer. A magnetic nanoparticle-based gene therapy pre-loaded with anti-piR-823, applied in a mouse xenograft model of human BC, effectively inhibited tumor initiation and growth [18]. In parallel, targeting DNMT3b might be a potential approach for cancer therapy. Many nucleoside and non-nucleoside inhibitors are validated to target several DNMTs, including DNMT3B in various cancers [52]. Targeting DNMT3B-mediated epigenetic reprogramming, either alone or in combination with piRNA-directed therapies, may therefore represent a potential avenue for future therapeutic development in ovarian cancer.

Numerous studies have demonstrated a strong association between EMT and cancer stemness in OC progression. OC cells often exhibit the expression of both EMT-related genes and stem cell markers, which contribute to metastasis and therapy resistance [53,54]. In our study, we observed a marked downregulation of CDH1 and concurrent upregulation of CDH2 in OC tissues, indicating a mesenchymal shift characteristic of EMT. CDH1 and CDH2 were canonical epithelial and mesenchymal markers, respectively, commonly used to evaluate EMT status and tumor aggressiveness [33]. Concomitantly, stemness-associated transcription factors OCT4 and NANOG were significantly upregulated, reinforcing the notion of enhanced stem-like features in these tumors. OCT4 and NANOG were considered key stemness-related transcription factors due to their well-established roles in maintaining pluripotency and cancer stem cell phenotypes in OC [55].

These findings support the interplay between EMT and cancer stemness in OC and suggest that both processes may be co-regulated, contributing synergistically to tumor progression. Our piR-823 target enrichment analysis revealed that piR-823 may modulate key biological processes, including epithelial development, cytoskeletal remodeling, and EMT signaling, potentially through the regulation of epigenetic modifiers. The analysis also highlighted PIK3R2 among the enriched pathways, a gene known for its critical role in stemness regulation [56,57]. These findings are consistent with previous reports demonstrating that piR-823 enhances stem-like properties in MM and BC cells [18,51], supporting its broader role in maintaining cancer stemness phenotypes.

DNA methylation is also a key molecular mechanism that regulates EMT and cancer stemness in OC, providing cellular plasticity for metastatic advantage and cancer stemness [30,58]. The Wnt signaling pathway is one of the most prominent pathways implicated in ovarian carcinogenesis. It plays a critical role in regulating CSCs’ characteristics and EMT processes, thereby contributing to the development and progression of various OC subtypes [59]. Emerging evidence suggests that aberrant activation of the Wnt signaling pathway can occur through multiple mechanisms, including the silencing of APC, a direct inhibitor of Wnt signaling [60,61]. Additionally, CDH1, a key epithelial marker and metastasis suppressor, influences Wnt signaling [62,63]. Notably, gene silencing caused by hypermethylation of CDH1 has been linked to the loss of CDH1 protein expression, leading to the activation of the canonical Wnt/β-catenin signaling pathway [28,64]. In this study, bioinformatics analysis revealed that the promoters of CDH1 were hypermethylated in OC samples, while DNMT3B was significantly expressed in Wnt-altered pathways. Functional enrichment analysis of piR-823 targets identified IQGAP1 [57], a scaffold protein that directly interacts with β-catenin, along with PIK3R2 [65], and TGFB2 [66], both of which are well-established modulators of Wnt signaling. Based on these findings, we propose that DNMT3B may contribute to hypermethylation-induced silencing of CDH1, thereby promoting stemness and EMT in OC through activation of the Wnt signaling pathway. However, this hypothesis warrants further experimental validation to confirm the proposed mechanisms.

**Figure 8:**
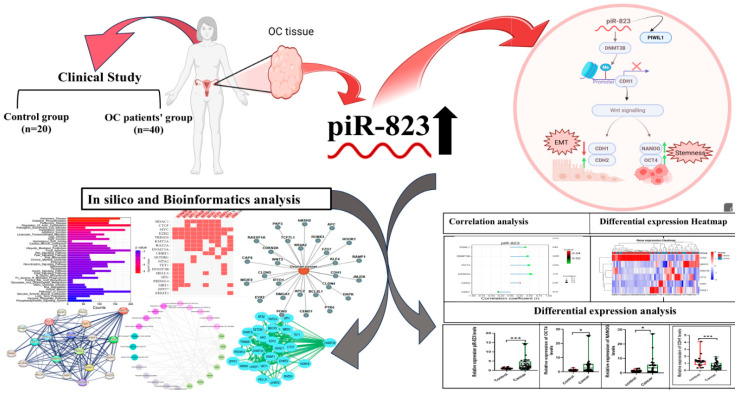
Integrated clinical and bioinformatics workflow illustrating the role of piR-823 in ovarian cancer (the study graphical abstract).

The PIWIL1 gene, a member of the PIWI subfamily of Argonaute proteins, plays a critical role in stem cell self-renewal, RNA silencing, and translational regulation across various organisms. As an oncogene, PIWIL1 has been reported to be overexpressed in several tumors, including endometrial, cervical, colon, ovarian, and hepatic cancer [67,68,69]. Moreover, PIWIL1 has been implicated in regulating EMT and promoting stem-like characteristics in endometrial cancer cells [70]. A previous study revealed a significant association of PIWIL1 and piR-823 in the tumor tissue of patients with RCC [24]. Consistently, our results showed a strong correlation between PIWIL1 and piR-823 expression levels in OC patients and different OC stratified groups, suggesting that PIWIL1 may regulate the biogenesis and function of piR-823. Taken together, our results suggest a novel regulatory axis of piR-823/PIWIL1/DNMT3B/CDH1 (Figure 8). This axis links non-coding RNA regulation with DNA methylation and transcriptional control of cell adhesion and stemness, providing mechanistic insight into OC metastasis and progression.

## 4. Materials and Methods

### 4.1. Subjects

#### 4.1.1. Sample Size and Power Calculation

Based on the previous study [20], the number of cases required to attain a type I error (*α* < 0.05) and a power of 95% is only about 50 samples using the G*power sample size online calculator (version 3.1.9.7) (http://www.gpower.hhu.de/en.html) (accessed on 16 May 2022). The required sample size was 50 and was increased by 20% to account for potential loss; the total sample was finally 60 subjects, 40 OC subjects and 20 controls (2:1).

#### 4.1.2. Patient Group

A total of 40 female patients, diagnosed with primary malignant ovarian tumors, were included in this study. All OC patients were treatment-naïve and were part of an Egyptian cohort admitted to the Gynecology and Obstetrics or Oncology Department at Ain Shams University Hospitals, Cairo, Egypt. OC tissue samples were obtained during surgical resection and confirmed through postoperative pathological examinations. Patients were enrolled only after meeting the inclusion criteria and providing written informed consent.

Inclusion criteria: this study included females aged 25–75 years, newly diagnosed, untreated cases of patients with histopathologically confirmed OC. Exclusion criteria: patients with a history of cancer other than OC, those with insufficient data or missing histological diagnosis, and patients undergoing chemotherapy and radiation therapy were excluded.

#### 4.1.3. Patients’ Demographic and Pathological Data

The patients’ demographic data, including age and menopausal status, and medical history were retrieved from hospital records. Along with original pathology reports documenting the following data (tumor site, tumor size, tumor type, lymph node metastasis (LNM), distant metastasis, tumor stage, ascites, and tumor grade) were obtained from the Department of Pathology, Ain Shams Hospitals. OC staging was performed based on the FIGO criteria, classifying tumors into stages I, II, III, and IV [71]. Tumor grading was determined based on the degree of cellular differentiation of OC, classifying OC into grade 1 (well differentiated), grade 2 (moderately differentiated), and grade 3 (poorly differentiated).

#### 4.1.4. Control Group

A total of 20 histopathologically confirmed normal ovarian tissues were collected from patients undergoing uterine and ovarian resection due to benign uterine diseases. The control group age ranged from 30 to 66 years.

### 4.2. Methods

#### 4.2.1. Tissue Samples

Fresh ovarian tissue samples were collected from study participants who underwent surgical resection at the Department of Obstetrics and Gynecology, Ain Shams University Hospitals. Multiple tissue sections were excised from different regions of both ovarian tumors and normal tissues during surgery. All collected tissue samples were immediately preserved in RNAlater stabilization solution (Cat. No. AM7020; Invitrogen, Life Technologies, Inc., Thermo Fisher Scientific, Fountain Drive, Inchinnan Business Park, Paisley, UK). The samples were kept at 2–8 °C overnight and subsequently stored at −20 °C for further quantitative analysis.

#### 4.2.2. Total RNA Extraction and Quantification

Total RNA was extracted from ovarian tissue samples using QIAzol lysis reagent (Cat. No. 79306; Qiagen, Hilden, Germany) and purified using the RNeasy Mini kit (Cat. No. 74104; Qiagen, Hilden, Germany) according to the manufacturer’s protocol. Briefly, approximately 100 mg of ovarian tissue was homogenized in liquid nitrogen, and QIAzol lysis reagent was added. After adding chloroform (Fisher Scientific, Thermo Fisher Scientific, Loughborough; Bishop Meadow Road, UK) and centrifuging, the mixture separated into two phases: a lower organic phase and an upper aqueous phase containing RNA. The upper aqueous phase was carefully transferred to the RNeasy Mini spin column for further purification. The purified RNA was eluted in 30 μL of RNase-free water, and its concentration and purity were assessed using a Denovix^®^ DS-11 spectrophotometer (Wilmington, DE, USA). The extracted RNA samples were stored at −80 °C until further analysis.

#### 4.2.3. cDNA Synthesis and Measurement of piR-823 Expression Using qRT-PCR

For piR-823 expression analysis, total RNA was reverse transcribed by miScript II Kit (Cat. No. 218161; Qiagen, Hilden, Germany) as per the manufacturer’s protocol. The resulting cDNA was stored at −20 °C until quantification. qRT-PCR analysis was subsequently performed using miRCURY LNA SYBR Green PCR Kit (Cat. No.339345; Qiagen, Hilden, Germany) as proposed by the manufacturer’s instructions using U6 as an endogenous control. The thermal cycling was performed using a two-step cycling protocol as follows: 2 min at 95 °C for initial activation, followed by 40 cycles of denaturation at 95 °C for 10 s, and combined annealing/extension at 56 °C for 60 s. The qRT-PCR was carried out using a Step-One Plus PCR detection system (Applied Biosystems, Foster City, CA, USA).

#### 4.2.4. cDNA Synthesis and Measurement of mRNA Expression of Target Genes Using qRT-PCR

For analyzing mRNA expression levels of target genes, RevertAid First Strand cDNA Synthesis Kit (Cat. No. K1622; Thermo Scientific, Rockford, IL, USA) was used as instructed by the manufacturer. The resulting cDNA was stored at −20 °C until the qRT-PCR process was carried out. For qRT-PCR assays, Maxima SYBR Green/ROX Master Mix (Cat. No. K0221; Thermo Scientific, Rockford, IL, USA) was used, with GAPDH as the endogenous control. The thermal cycling conditions were performed using a three-step cycling protocol as follows: 10 min at 95 °C for activation, followed by 40 cycles of denaturation at 95 °C for 15 s, 60 °C for 30 s, and 72 °C for 30 s. The qRT-PCR was carried out using a Step-One Plus PCR detection system (Applied Biosystems, Foster City, CA, USA). qRT-PCR experiments were performed in technical duplicates for each sample, and average Ct values were used for analysis. All samples were processed under identical experimental conditions using the same reagents and protocols, and gene expression levels were normalized to endogenous reference to control for the potential batch effects.

All primers used in the analysis are listed in Table 8. The RNA relative expression levels were computed and normalized as fold change using the cycle threshold (Ct) method (2^−ΔΔCt^).

#### 4.2.5. In Silico Database Search and Bioinformatics Analysis (Accessed on 16 January 2023)

Sequences of the target genes were retrieved from the Ensembl genome database (https://www.ensembl.org/index.html). Primer pairs with optimal scores were designed using the Primer3 tool (https://primer3.ut.ee/). To ensure specificity and avoid off-target amplification, the designed primers were validated through UCSC In Silico PCR (https://genome.ucsc.edu/cgi-bin/hgPcr), BLAST (https://blast.ncbi.nlm.nih.gov/Blast.cgi), and IDT Oligoanalyzer (https://eu.idtdna.com/pages/tools/oligoanalyzer?returnurl=%2Fcalc%2Fanalyzer).

The differential expression of the investigated markers between primary OC tissues and normal tissues was further analyzed using the GEPIA database (http://gepia.cancer-pku.cn/detail.php), UALCAN database (https://ualcan.path.uab.edu/analysis-surv.html) and TNM plot database. Survival analysis of the investigated markers was visualized using the KM-Plot database (http://www.kmplot.com/) and GENT2 database (http://gent2.appex.kr/gent2/).

Protein–protein interaction (PPI) networks involving DNMT3B were generated using the STRING (https://string-db.org/) and INDRA databases (http://indra.bio). Functional enrichment analysis of DNMT3B interactions were performed using the Enrichr tool (https://maayanlab.cloud/Enrichr/#find!gene=PAK2) and StarBase database (https://rnasysu.com/encori/) to explore DNMT3B-related pathways and biological processes in OC. The MethBank database (https://ngdc.cncb.ac.cn/methbank/) used to analyze differentially methylated genes in OC. Putative piR-823 target genes were identified using the sRNAtools database (https://rnainformatics.org.cn/sRNAtools/index.php). Target prediction was performed using the miRanda algorithm with a score threshold >160 and an energy threshold <–20 kcal/mol. The predicted targets were subsequently subjected to functional enrichment analysis using Enrichr-KG (https://maayanlab.cloud/enrichr-kg) to identify significantly associated biological pathways.

#### 4.2.6. Statistical Analysis

Data were tested for normality using the Shapiro–Wilk normality test. Data were expressed as median and interquartile range (IQR: 25th–75th percentile) for non-parametric data and as mean ± standard error of the mean (S.E.M.) for parametric data. Comparative analyses included the Mann–Whitney U test for non-parametric comparisons between two groups, while the independent sample *t*-test was applied for parametric data comparisons. For comparisons involving more than two groups, the Kruskal–Wallis test was performed. Correlation analysis was conducted using Spearman’s correlation coefficient (*r*) to assess associations between continuous variables. Point biserial correlation was used to evaluate relationships involving dichotomous variables. The Chi-square test was performed to examine the association between piR-823 expression and clinicopathological features in OC samples. Any skewed data were logarithmically transformed before performing simple and multiple linear regression analyses to explore the association between piR-823 and each of the downstream mRNA targets DNMT3B, OCT4, NANOG, CDH1, and CDH2. In multiple regression models, piR-823 and DNMT3B served as independent variables, while OCT4, NANOG, CDH2, and CDH1 were the dependent variables. Statistical significance was defined as *p* < 0.05 (two-tailed). Statistical analyses were conducted using SPSS 27.0 software (https://www.ibm.com/products/spss-statistics) (accessed on 16 July 2024). Graphs were generated using GraphPad Prism 8.0 (https://www.graphpad.com/scientific-software/prism/) (accessed on 16 July 2024).

## 5. Conclusions

In this study, we explored the expression and oncogenic role of piR-823 in OC tissue samples and its association with epigenetic regulation, cancer stemness, and EMT. Our findings revealed that piR-823 is significantly overexpressed in OC tissues and correlates with poor prognostic indicators, including LNM, tumor grade, and FIGO staging. Furthermore, piR-823 demonstrated potential as a diagnostic biomarker, with high sensitivity and specificity, as indicated by ROC curve analysis. piR-823 appears to promote OC progression through interactions with DNMT3B, underscoring DNMT3B’s role in epigenetically regulating stemness and EMT-related markers. This study proposed a putative axis of piR-823/PIWIL1/DNMT3B/CDH1 that appears to be involved in EMT and cancer stemness in OC, providing a basis for future mechanistic studies. These findings highlight piR-823 as a promising diagnostic and prognostic biomarker and a potential therapeutic target in OC.

Limitations and Future Perspectives. Although bioinformatics and correlation analyses suggest a role for piR-823 in OC progression, future functional studies using piR-823 knockdown or inhibition in OC cells will be essential to validate the mechanistic role of the proposed piR-823/PIWIL1/DNMT3B/CDH1 axis.

In line with the growing interest in ncRNA-based stratification and biomarker-driven precision oncology, previous clinical and in silico studies have demonstrated the utility of integrated molecular signatures in cancer classification, prognosis, therapeutic targeting of genetic and/or epigenetic regulators in relation to obesity or diabetes or other chronic diseases, genomic variants or single-nucleotide polymorphisms, which require further investigation to establish detailed mechanistic precision pathways [72,73,74,75,76]. The current findings serve as a stepping-stone for deeper investigations into the exploration and development of piRNA-823-based gene therapy as a novel therapeutic strategy using non-coding genomes to suppress OC, advancing ncRNA precision. Moreover, the study of piR in relation to DNA damage [77] or telomere length [78], as well as the effect of immunomodulators on their expression level with other ncRNAs including ncRNA–miRNA regulatory axes or signaling pathways, inflammatory, and/or metabolic biomarkers [79,80,81], respectively, may further support the clinical translation of piRNA-based biomarker use in cancer and/or chronic disease research, a step-toward precision.

## Figures and Tables

**Table 1:** Demographic, clinical, and pathological parameters/characteristics of study participants (n = 60).

Parameter	Control (n = 20)	OC (n = 40)	*p.* Value
Age (years)	53.35 ± 2.03	53.55 ± 1.77	χ^2^ = 0.35 *p*1 = 0.55 *p*2 = 0.47
≥50 (n, %)	13 (65%)	29 (72.5%)	
<50 (n, %)	7 (35%)	11 (27.5%)	
Menopause state			χ^2^ = 0.83 *p*1 = 0.36
Pre (n, %)	8 (52.5%)	19 (52.5%)	
post (n, %)	12 (47.5%)	21 (47.5%)	
piR-823 fold expression	1.00 (0.818–1.381)	2.41 (1.312–4.691)	*p*2 < 0.001
PIWIL1 fold expression	1.38 (1.136–1.611)	3.61 (1.138–4.775)	*p*2 = 0.009
DNMT3B fold expression	1.21 (0.721–1.483)	4.04 (1.669–6.145)	*p*2 < 0.001
OCT4 fold expression	1.22 (0.841–1.569)	2.63 (1.051–5.547)	*p*2 = 0.011
NANOG fold expression	1.28 (0.679–1.680)	3.44 (0.787–5.640)	*p*2 = 0.03
CDH2 fold expression	1.27 (0.936–1.665)	19.3 (1.329–32.792)	*p*2 < 0.001
CDH1 fold expression	1.26 (0.789–1.404)	0.59 (0.238–1.042)	*p*2 < 0.001
Tumor type			
Serous (n, %)	-	2.53 (1.01–3.54)	N.A.
Others (n, %)	-	2.33 (1.32–5.53)	N.A.
Tumor site			
Unilateral (n, %)	-	22 (55%)	N.A.
Bilateral (n, %)	-	18 (45%)	N.A.
Tumor size			
≥10 cm (n, %)	-	29 (72.5%)	N.A.
<10 (n, %)	-	11 (27.5%)	N.A.
FIGO stage			
T I, II (n, %)	-	19 (47.5%)	N.A.
T III, IV (n, %)	-	21 (52.5%)	N.A.
Histological grade			
GI, GII (n, %)	-	18 (45%)	N.A.
GIII (n, %)	-	22 (55%)	N.A.
LNM			
Absent (n, %)	-	20 (50%)	N.A.
Present (n, %)	-	20 (50%)	N.A.
Distant Metastasis			
Absent (n, %)	-	33 (82.5%)	N.A.
Present (n, %)	-	7 (17.5%)	N.A.
Ascites			
Absent (n, %)	-	19 (47.5%)	N.A.
Present (n, %)	-	21 (52.5%)	N.A.

Results are represented as median (25th quartile–75th quartile) for non-parametric data and mean ± (S.E.M.) for parametric data (age). Statistics were computed using Chi-square test χ^2^ (dichotomous parameters), Student’s *t* test (parametric data), and Mann–Whitney test (non-parametric data), *p*1 denotes comparison between number of participants in control and OC groups using Chi-square test, while *p*2 denotes comparison between median or mean of control and OC group. [DNMT3B, DNA methyltransferase 3B; CDH1, E-cadherin; LNM, lymph node metastasis; CDH2, N-cadherin; OCT4, octamer-binding transcription factor 4; PIWIL1, piwi-like RNA-mediated gene silencing 1; N.A., non-available].

**Table 2:** Expression of piR-823 and investigated molecular biomarkers targets in OC patient (n = 40) stratified groups.

Characteristic		n (%)	piR-823	PIWIL1	DNMT3B	OCT4	NANOG	CDH2	CDH1
Age (years)	≥50	29 (72.5%)	2.8 (1.11–5.04)	3.12 (0.98–4.81)	3.96 (1.67–5.22)	1.63 (1.01–5.38)	2.74 (0.77–6.21)	19.8 (1.71–29.25)	0.46 (0.21–1.03)
	<50	11 (27.5%)	1.92 (1.34–2.66)	4.40 (1.23–4.7)	4.11 (1.65–12.1)	4.58 (1.58–7.31)	3.66 (0.77–5.53)	18.80 (1.10–50)	0.68 (0.56–1.19)
	*p*-value		NS	NS	NS	NS	NS	NS	NS
Menopausal state	Pre-	21 (52.5%)	2.50 (1.60–5.56)	4.4 (1.27–7.16)	4.46 (2.36–7.62)	4.58 (1.36–6.23)	5.03 (1.31–6.21)	17.81 (1.34–29.25)	0.66 (0.33–1.1)
	Post	19 (47.5%)	2.1 (0.87–3.58)	2.72 (0.86–4.6)	2.82 (0.75–4.81)	1.39 (0.98–4.44)	1.11 (0.4–5.35)	19.82 (0.87–46.52)	0.42 (0.21–0.86)
	*p*-value		NS	NS	NS	NS	NS	NS	NS
Tumor type	Serous	15 (37.5%)	2.53 (1.01–3.54)	3.12 (0.62–4.5)	4.41 (0.97–6.39)	4.44 (1.24–5.68)	5.13 (0.94–5.48)	2.46 (0.24–23.91)	0.66 (0.37–1.12)
	Others	25 (62.5%)	2.33 (1.32–5.53)	4.39 (1.27–4.81)	3.96 (2.01–5.39)	1.64 (0.98–5.33)	2.31 (0.71–6.98)	19.8 (2.86–39.82)	0.56 (0.19–0.93)
	*p*-value		NS	NS	NS	NS	NS	NS	NS
Tumor site	Unilateral	22 (55%)	2.21 (1.29–5.39)	2.68 (1.21–4.62)	3.84 (1.73–5.73)	1.54 (0.87–4.38)	1.4 (0.81–5.35)	19.95 (2.92–47.39)	0.51 (0.19–0.78)
	Bilateral	18 (45%)	2.59 (1.26–4.61)	4.20 (0.77–7.12)	4.31 (1.48–6.39)	4.81 (1.26–25.18)	5.24 (0.71–7.26)	3.13 (0.27–24.61)	0.68 (0.35–1.16)
	*p*-value		NS	NS	NS	NS	NS	NS	NS
Tumor size	≥10 cm	29 (72.5%)	2.50 (1.31–5.43)	4.3 (0.98–4.65)	4.11 (1.97–4.94)	2.53 (1.05–5.43)	2.74 (0.74–5.48)	19.8 (1.69–36.6)	0.61 (0.33–1.03)
	<10	11 (27.5%)	2.10 (1.30–3.44)	3 (1.23–9.48)	3.96 (0.58–7.92)	4.08 (0.26–5.88)	5.13 (1.36–7.33)	11.23 (1.31–33.1)	0.42 (0.11–1.19)
	*p*-value		NS	NS	NS	NS	NS	NS	NS
FIGO stage	T I, II	19 (47.5%)	1.78 (0.87–2.66)	3.12 (0.86–4.5)	4.11 (1.67–7.92)	1.63 (0.74–6.58)	2.31 (0.71–5.67)	17.81 (1.1–31.77)	0.56 (0.2–1.02)
	T III, IV	21 (52.5%)	3.44 (1.75–5.56)	4.1 (1.17–7.16)	3.96 (1.66–5.39)	3.73 (1.33–5.52)	4.93 (1.07–6.14)	19.82 (1.77–39.82)	0.61 (0.29–1.08)
	*p*-value		0.027	NS	NS	NS	NS	NS	NS
Histological grade	GI, GII	18 (45%)	1.60 (0.86–3.49)	1.76 (0.81–4.41)	3.27 (0.69–6.36)	1.16 (0.64–3.37)	1.23 (0.75–5.94)	18.8 (0.85–35.46)	0.68 (0.2–1.11)
	GIII	22 (55%)	3.07 (1.81–5.56)	4.49 (1.32–6.04)	4.16 (2.14–5.74)	4.51 (1.05–6.05)	5.08 (1.07–5.89)	19.31 (1.96–37.34)	0.56 (0.35–0.91)
	*p*-value		0.022	NS	NS	0.012	NS	NS	NS
LNM	Absent	20 (50%)	1.42 (0.84–4.09)	2.68 (0.69–4.57)	2.98 (1.15–6.02)	1.64 (0.8–5.35)	2.98 (0.61–5.63)	18.23 (1.15–50)	0.56 (0.23–0.79)
	Present	20 (50%)	3.39 (1.99–5.54)	4.44 (1.31–8.32)	4.44 (1.68–6.14)	3.91 (1.31–5.65)	4.07 (0.88–6.44)	19.3 (1.61–26)	0.63 (0.25–1.11)
	*p*-value		0.02	NS	NS	NS	NS	NS	NS
Distant Metastasis	Absent	33 (82.5%)	1.6 (1.01–2.81)	4.3 (1.27–4.76)	4.11 (1.67–5.94)	1.64 (1.01–5.52)	5.03 (0.95–6.21)	17.81 (1.04–24.75)	0.56 (0.20–1.05)
	Present	7 (17.5%)	2.5 (1.34–5.20)	1.32 (0.62–4.8)	3.96 (1.65–8.85)	3.73 (1.58–5.68)	1.06 (0.32–3.21)	33.13 (2.29–50)	0.71 (0.42–1.04)
	*p*-value		NS	NS	NS	NS	NS	NS	NS
Ascites	Absent	19 (47.5%)	3.07 (1.93–5.54)	1.32 (0.62–4.6)	3.15 (0.75–6.39)	1.39 (0.48–4.08)	1.08 (0.34–5.53)	11.23 (0.77–33.13)	0.66 (0.34–1.19)
	Present	21 (52.5%)	1.63 (0.84–3.47)	4.39 (2.01–7.16)	4.46 (2.1–5.39)	5.03 (1.36–5.78)	5.13 (1.27–6.86)	20.11 (2.37–40.88)	0.56 (0.2–0.95)
	*p*-value		0.02	NS		0.023	0.02	NS	NS
Menopausal state	Pre-	21 (52.5%)	2.50 (1.60–5.56)	4.4 (1.27–7.16)	4.46 (2.36–7.62)	4.58 (1.36–6.23)	5.03 (1.31–6.21)	17.81 (1.34–29.25)	0.66 (0.33–1.1)
	Post	19 (47.5%)	2.1 (0.87–3.58)	2.72 (0.86–4.6)	2.82 (0.75–4.81)	1.39 (0.98–4.44)	1.11 (0.4–5.35)	19.82 (0.87–46.52)	0.42 (0.21–0.86)
	*p*-value		NS	NS	NS	NS	NS	NS	NS

All data are presented as median (IQR: 25th–75th percentile). Statistics were computed using Mann–Whitney test. *p*-values < 0.05 were considered statistically significant. [DNMT3B, DNA methyltransferase 3B; CDH1, E-cadherin; LNM, lymph node metastasis; CDH2, N-cadherin; OCT4, octamer-binding transcription factor 4; PIWIL1, piwi-like RNA-mediated gene silencing 1; NS, non-significant].

**Table 3:** Correlation of piR-823, PIWIL1, DNMT3B, OCT4, NANOG, CDH2 and CDH1 expression levels with demographic and clinicopathological features within OC patients (n = 40).

Subgroup	Correlation Coefficient	piR-823	PIWIL1	DNMT3B	OCT4	NANOG	CDH2	CDH1
Age (years)	Spearman	−0.079	−0.030	−0.234	−0.157	−0.162	0.073	−0.107
Menopausal state	Point-Biserial	−0.287	−0.173	−0.281	−0.133	−0.104	0.054	−0.071
Tumor type	Point-Biserial	−0.182	−0.082	0.013	0.185	0.085	−0.192	0.152
Tumor site	Point-Biserial	−0.024	0.133	0.009	0.432 **	0.232	−0.218	0.188
Tumor size (cm)	Spearman	−0.099	−0.086	0.033	−0.122	−0.383 *	0.123	0.095
FIGO stage	Point-Biserial	0.242	0.128	−0.173	−0.043	−0.074	0.025	−0.041
Grade	Point-Biserial	0.348 *	0.211	0.107	0.272	0.023	0.029	−0.092
LNM	Point-Biserial	0.119	0.207	0.054	−0.010	−0.072	−0.094	0.034
Distant metastasis	Point-Biserial	−0.182	−0.094	0.044	0.039	−0.208	0.255	0.088
Ascites	Point-Biserial	0.237	0.209	0.031	0.266	0.148	0.132	−0.177

Spearman correlation coefficient *r* test was used to measure continuous non-parametric variables, while point-biserial correlation was used to measure the association that exists between two variables; one continuous and the other is dichotomous using SPSS software. * Significant statistical difference *p*-value < 0.05. ** Significant statistical difference *p*-value < 0.01. [LNM, lymph node metastasis; DNMT3B, DNA methyltransferase 3B; CDH1, E-cadherin; CDH2, N-cadherin; OCT4, octamer-binding transcription factor 4; PIWIL1, piwi-like RNA-mediated gene silencing 1].

**Table 4:** Correlation coefficients (*r*) and *p*-values of piR-823, PIWIL1, DNMT3B, OCT4, NANOG, CDH2 and CDH1 levels in OC patients (n = 40) and control (n = 20).

	Correlation Coefficient	piR-823	PIWIL1	DNMT3B	OCT4	NANOG	CDH2	CDH1
piR-823	*r*	1.000	0.391 **	0.451 ***	0.479 ***	0.533 ***	0.257 *	−0.315 *
PIWIL1	*r*	0.391 **	1.000	0.351 **	0.436 ***	0.42 ***	0.208	0.024
DNMT3B	*r*	0.451 ***	0.351 **	1.000	0.450 ***	0.389 **	0.517 ***	−0.308 *
OCT4	*r*	0.479 ***	0.436 ***	0.450 ***	1.000	0.346 **	0.252	−0.158
NANOG	*r*	0.533 ***	0.42 ***	0.389 **	0.346 **	1.000	0.026	−0.231
CDH2	*r*	0.257 *	0.208	0.517 ***	0.252	0.026	1.000	−0.207
CDH1	*r*	−0.315 *	0.024	−0.308 *	−0.158	−0.231	−0.207	1.000

Correlation coefficient was calculated using SPSS software and determined by Spearman correlation *r* test. * Statistical significance *p*-value < 0.05. ** Statistical significance is at *p*-value < 0.01. *** Statistical significance is at *p*-value < 0.001. [DNMT3B, DNA methyltransferase 3B; CDH1, E-cadherin; CDH2, N-cadherin; OCT4, octamer-binding transcription factor 4; PIWIL1, piwi-like RNA-mediated gene silencing 1].

**Table 5:** Spearman correlation coefficient of piR-823 combined with other mRNA expression in OC group (n = 40) and various OC subgroups N (%).

Subgroup	N (%)	piR-823/ PIWIL1	piR-823/ DNMT3B	piR-823/ OCT4	piR-823/ NANOG	piR-823/ CDH2	piR-823/ CDH1
OC	40 (100%)	0.402 *	0.141	0.421 **	0.405 **	0.014	−0.090
Age ≥ 50	29 (72.5%)	0.514 **	0.320	0.400 *	0.490 **	0.033	0.060
Serous subtype	15 (37.5%)	0.393	0.475	0.664 **	0.432	0.547 *	−0.036
Bilateral tumor	18 (45%)	0.418	0.351	0.602 **	0.302	0.508 *	0.146
Tumor size > 10 cm	29 (72.5%)	0.285	0.195	0.332	0.451 *	−0.018	−0.162
FIGO stage III/IV	21 (52.5%)	0.595 **	0.103	0.125	0.427	−0.040	0.212
Grade III	22 (55%)	0.249	−0.011	0.400	0.144	0.056	0.082
Positive LNM	20 (50%)	0.430	−0.141	−0.069	0.341	−0.133	0.120
Positive Distant Metastasis	7 (17.5%)	0.679	0.464	0.750	0.429	−0.111	0.357
Positive Ascites	21(52.5%)	0.482 *	−0.119	0.184	0.105	−0.094	0.255
Post-menopausal	19 (47.5%)	0.319	0.409	0.341	0.733 ***	0.054	−0.263

Spearman correlation coefficient *r* test was calculated using SPSS software, * Statistical significance *p*-value < 0.05. ** Statistical significance is at *p*-value < 0.01. *** Statistical significance is at *p*-value < 0.001. [LNM, lymph node metastasis; DNMT3B, DNA methyltransferase 3B; CDH1, E-cadherin; CDH2, N-cadherin; OCT4, octamer-binding transcription factor 4; PIWIL1, piwi-like RNA-mediated gene silencing 1].

**Table 6:** Simple Linear Regression Analysis of piR-823 and DNMT3B as Independent Variables in Relation to Investigated Molecular Biomarkers.

Dependent Variables	Independent Variables							
	piR-823 ^a^				DNMT3B ^a^			
	R^2^	B	CI	*p*-Value	R^2^	B	CI	*p*-Value
DNMT3B ^a^	0.158	0.46	[0.18–0.73]	0.002	-			
OCT4 ^a^	0.21	0.63	[0.32–0.95]	<0.001	0.17	0.48	[0.20–0.76]	0.001
NANOG ^a^	0.258	0.69	[0.38–1.00]	<0.001	0.16	0.47	[0.19–0.76]	0.001
CDH2 ^a^	0.072	0.56	[0.034–1.10]	0.038	0.253	0.92	[0.50–1.33]	<0.001
CDH1 ^a^	0.093	−0.31	[−0.57–0.057]	0.018	0.07	−0.23	[−0.46–0.009]	0.042

^a^ Logarithmically transformed values were used.

**Table 7:** Multiple linear regression analysis for stemness and EMT related genes.

Dependent Variables	Model Coefficients	Predictors	Unstandardized Coefficients		Std β	95% CI	*p*-Value
			B	Std. Error			
OCT4 ^a^	*p* = 0.0003 R^2^ = 0.28	piR-823 ^a^	0.49	0.166	0.36	[0.158–0.825]	0.005
		DNMT3B ^a^	0.31	0.144	0.26	[0.027–0.604]	0.03
NANOG ^a^	*p* < 0.0001 R^2^ = 0.30	piR-823 ^a^	0.56	0.164	0.41	[0.235–0.983]	0.001
		DNMT3B	0.28	0.142	0.24	[−0.001–0.569]	NS
CDH2 ^a^	*p* < 0.001 R^2^= 0.259	piR-823 ^a^	1.72	0.262	0.08	[−0.353–0.689]	NS
		DNMT3B ^a^	0.86	0.227	0.47	[0.406–1.317]	0.001
CDH1 ^a^	*p* = 0.028 R^2^ = 0.11	piR-823 ^a^	−0.246	0.140	−0.238	[−5.27–0.034]	NS
		DNMT3B ^a^	−0.151	0.121	−1.69	[−3.94–0.092]	0.21

^a^ Logarithmically transformed values were used. [NS, non-significant.]

**Table 8:** Primer sequence for the study.

Primer	Forward (5′–3′)	Reverse (5′–3′)
piR-823	AGCGTTGGTGGTATAGTGGT	N/A
PIWIL1	GTCTGTTGTCAAGTAATCGGAAGG	TTGCTGTTTGCCTAAGGTTCG
DNMT3B	CATGGTGGTGTCTTGGAAGG	TGGCTTTTCGATAGGAGACG
OCT4	TGGAGAAGGAGAAGCTGGAG	TGCAGTGTGGGTTTCGGG
NANOG	TTGTGGGCCTGAAGAAAACT	TTTGCGACACTCTTCTCTGC
CDH2	AGGTGGAGGAGAAGAAGACC	CATTCGTCGGATTCCCACAG
CDH1	CCCATCTTTGTGCCTCCTGA	CGTGCTCAAAATCCTCCCTG
GAPDH	AGCCACATCGCTCAGACAC	GCCCAATACGACCAAATCC
U6	ATTGGAACGATACAGAGAAGATT	GGAACGCTTCACGAATTTG

[N/A: not available for researchers, intellectual property of Qiagen Co.].

## Data Availability

The original contributions presented in this study are included in the article/Supplementary Materials. Further inquiries can be directed to the corresponding authors.

## Supplementary Materials

The following supporting information can be downloaded at: https://www.mdpi.com/article/10.3390/ijms27020823/s1.

## Abbreviations

APC	adenomatous polyposis coli
BC	breast cancer
CRC	colorectal cancer
CSCs	cancer stem cells
DNMT3B	DNA methyltransferase 3B
CDH1	E-cadherin
EMT	epithelial–mesenchymal transition
ESCC	esophageal squamous cell carcinoma
FIGO	International Federation of Gynecology and Obstetrics
GO	Gene Ontology
GAPDH	Glyceraldehyde 3 Phosphate Dehydrogenase
KEGG	Kyoto Encyclopedia of Genes and Genomes
LNM	lymph node metastasis
MM	Multiple myeloma
CDH2	N-cadherin
OCT4	Octamer-binding transcription factor 4
OC	ovarian cancer
PIWIL1	piwi-like RNA-mediated gene silencing 1
piRNAs	P-element-induced wimpy testis (PIWI)-interacting RNAs
PPI	protein–protein interaction
RCC	renal cancer
Wnt	Wingless-related integration site

## References

[1] Yan Y., Tian D., Zhao B., Li Z., Huang Z., Li K., Chen X., Zhou L., Feng Y., Yang Z. (2024). piR-1919609 Is an Ideal Potential Target for Reversing Platinum Resistance in Ovarian Cancer. Technol. Cancer Res. Treat..

[2] Hassan A.A., Artemenko M., Tang M.K.S., Shi Z., Chen L.-Y., Lai H.-C., Yang Z., Shum H.-C., Wong A.S.T. (2022). Ascitic fluid shear stress in concert with hepatocyte growth factor drive stemness and chemoresistance of ovarian cancer cells via the c-Met-PI3K/Akt-miR-199a-3p signaling pathway. Cell Death Dis..

[3] Muinao T., Pal M., Deka Boruah H.P. (2018). Origins based clinical and molecular complexities of epithelial ovarian cancer. Int. J. Biol. Macromol..

[4] Pignata S., Cecere S.C., Du Bois A., Harter P., Heitz F. (2017). Treatment of recurrent ovarian cancer. Ann. Oncol..

[5] Reid B.M., Fridley B.L. (2020). DNA Methylation in Ovarian Cancer Susceptibility. Cancers.

[6] Beg A., Parveen R., Fouad H., Yahia M.E., Hassanein A.S. (2022). Role of different non-coding RNAs as ovarian cancer biomarkers. J. Ovarian Res..

[7] Motohara T., Yoshida G.J., Katabuchi H. (2021). The hallmarks of ovarian cancer stem cells and niches: Exploring their harmonious interplay in therapy resistance. Semin. Cancer Biol..

[8] Takai M., Terai Y., Kawaguchi H., Ashihara K., Fujiwara S., Tanaka T., Tsunetoh S., Tanaka Y., Sasaki H., Kanemura M. (2014). The EMT (epithelial-mesenchymal-transition)-related protein expression indicates the metastatic status and prognosis in patients with ovarian cancer. J. Ovarian Res..

[9] Elanany M.M., Mostafa D., Hamdy N.M. (2023). Remodeled tumor immune microenvironment (TIME) parade via natural killer cells reprogramming in breast cancer. Life Sci..

[10] Nowicki A., Kulus M., Wieczorkiewicz M., Pieńkowski W., Stefańska K., Skupin-Mrugalska P., Bryl R., Mozdziak P., Kempisty B., Piotrowska-Kempisty H. (2021). Ovarian Cancer and Cancer Stem Cells—Cellular and Molecular Characteristics, Signaling Pathways, and Usefulness as a Diagnostic Tool in Medicine and Oncology. Cancers.

[11] Motohara T., Masuda K., Morotti M., Zheng Y., El-Sahhar S., Chong K.Y., Wietek N., Alsaadi A., Carrami E.M., Hu Z. (2019). An evolving story of the metastatic voyage of ovarian cancer cells: Cellular and molecular orchestration of the adipose-rich metastatic microenvironment. Oncogene.

[12] Kumari A., Shonibare Z., Monavarian M., Arend R.C., Lee N.Y., Inman G.J., Mythreye K. (2021). TGFβ signaling networks in ovarian cancer progression and plasticity. Clin. Exp. Metastasis.

[13] Sato K., Siomi M.C. (2020). The piRNA pathway in Drosophila ovarian germ and somatic cells. Proc. Jpn. Acad. Ser. B.

[14] Garcia-Borja E., Siegl F., Mateu R., Slaby O., Sedo A., Busek P., Sana J. (2024). Critical appraisal of the piRNA-PIWI axis in cancer and cancer stem cells. Biomark. Res..

[15] Chattopadhyay T., Biswal P., Lalruatfela A., Mallick B. (2022). Emerging roles of PIWI-interacting RNAs (piRNAs) and PIWI proteins in head and neck cancer and their potential clinical implications. Biochim. Biophys. Acta (BBA)—Rev. Cancer.

[16] Guo B., Li D., Du L., Zhu X. (2020). piRNAs: Biogenesis and their potential roles in cancer. Cancer Metastasis Rev..

[17] Assumpção C.B., Calcagno D.Q., Araújo T.M.T., Batista dos Santos S.E., Ribeiro dos Santos Â.K.C., Riggins G.J., Burbano R.R., Assumpção P.P. (2015). The role of piRNA and its potential clinical implications in cancer. Epigenomics.

[18] Ding X., Li Y., Lü J., Zhao Q., Guo Y., Lu Z., Ma W., Liu P., Pestell R.G., Liang C. (2021). piRNA-823 Is Involved in Cancer Stem Cell Regulation Through Altering DNA Methylation in Association with Luminal Breast Cancer. Front. Cell Dev. Biol..

[19] Yin J., Jiang X., Qi W., Ji C., Xie X., Zhang D., Cui Z., Wang C., Bai Y., Wang J. (2017). piR-823 contributes to colorectal tumorigenesis by enhancing the transcriptional activity of HSF_1_. Cancer Sci..

[20] Feng J., Yang M., Wei Q., Song F., Zhang Y., Wang X., Liu B., Li J. (2020). Novel evidence for oncogenic piRNA-823 as a promising prognostic biomarker and a potential therapeutic target in colorectal cancer. J. Cell Mol. Med..

[21] Cheng J., Deng H., Xiao B., Zhou H., Zhou F., Shen Z., Guo J. (2012). piR-823, a novel non-coding small RNA, demonstrates in vitro and in vivo tumor suppressive activity in human gastric cancer cells. Cancer Lett..

[22] Su J.-F., Zhao F., Gao Z.-W., Hou Y.-J., Li Y.-Y., Duan L.-J., Lun S.-M., Yang H.-J., Li J.-K., Dai N.-T. (2020). piR-823 demonstrates tumor oncogenic activity in esophageal squamous cell carcinoma through DNA methylation induction via DNA methyltransferase 3B. Pathol. Res. Pract..

[23] Iliev R., Fedorko M., Machackova T., Mlcochova H., Svoboda M., Pacik D., Dolezel J., Stanik M., Slaby O. (2016). Expression Levels of PIWI-interacting RNA, piR-823, Are Deregulated in Tumor Tissue, Blood Serum and Urine of Patients with Renal Cell Carcinoma. Anticancer. Res..

[24] Slaby O., Iliev R., Stanik M., Fedorko M., Poprach A., Vychytilova-Faltejskova P., Slaba K., Svoboda M., Fabian P., Pacik D. (2016). Decreased expression levels of PIWIL1, PIWIL2, and PIWIL4 are associated with worse survival in renal cell carcinoma patients. Onco Targets Ther..

[25] Yan H., Wu Q.-L., Sun C.-Y., Ai L.-S., Deng J., Zhang L., Chen L., Chu Z.-B., Tang B., Wang K. (2015). piRNA-823 contributes to tumorigenesis by regulating de novo DNA methylation and angiogenesis in multiple myeloma. Leukemia.

[26] Li B., Hong J., Hong M., Wang Y., Yu T., Zang S., Wu Q. (2019). piRNA-823 delivered by multiple myeloma-derived extracellular vesicles promoted tumorigenesis through re-educating endothelial cells in the tumor environment. Oncogene.

[27] Xu K., Chen B., Li B., Li C., Zhang Y., Jiang N., Lang B. (2020). DNMT3B silencing suppresses migration and invasion by epigenetically promoting miR-34a in bladder cancer. Aging.

[28] Prasad C.P., Mirza S., Sharma G., Prashad R., DattaGupta S., Rath G., Ralhan R. (2008). Epigenetic alterations of CDH1 and APC genes: Relationship with activation of Wnt/β-catenin Pathway in invasive ductal carcinoma of breast. Life Sci..

[29] Rajabi H., Tagde A., Alam M., Bouillez A., Pitroda S., Suzuki Y., Kufe D. (2016). DNA methylation by DNMT1 and DNMT3b methyltransferases is driven by the MUC1-C oncoprotein in human carcinoma cells. Oncogene.

[30] Ye Z., Jiang Y., Wu J. (2022). DNMT3B attenuated the inhibition of TET3 on epithelial-mesenchymal transition in TGF-β1-induced ovarian cancer by methylating the TET3 promoter. Reprod. Biol..

[31] Papakonstantinou E., Pappa I., Androutsopoulos G., Adonakis G., Maroulis I., Tzelepi V. (2023). Comprehensive Analysis of DNA Methyltransferases Expression in Primary and Relapsed Ovarian Carcinoma. Cancers.

[32] Foster R., Buckanovich R.J., Rueda B.R. (2013). Ovarian cancer stem cells: Working towards the root of stemness. Cancer Lett..

[33] Teeuwssen M., Fodde R. (2019). Wnt Signaling in Ovarian Cancer Stemness, EMT, and Therapy Resistance. J. Clin. Med..

[34] Arend R.C., Londoño-Joshi A.I., Straughn J.M., Buchsbaum D.J. (2013). The Wnt/β-Catenin Pathway in Ovarian Cancer: A Review. Gynecol. Oncol..

[35] Qin H., Han Y., Li J., Wu Q., Du Y., Li Q., Chen X., Wang Y., Guan X., Sheng X. (2025). piRNA28846 has the potential to be a novel RNA nucleic acid drug for ovarian cancer. npj Precis. Oncol..

[36] Oner C., Colak E. (2022). PIWI Interacting RNA-823: Epigenetic Regulator of The Triple Negative Breast Cancer Cells Proliferation. Eurasian J. Med. Oncol..

[37] Sabbah N.A., Abdalla W.M., Mawla W.A., AbdAlMonem N., Gharib A.F., Abdul-Saboor A., Abdelazem A.S., Raafat N. (2021). piRNA-823 Is a Unique Potential Diagnostic Non-Invasive Biomarker in Colorectal Cancer Patients. Genes.

[38] Wang S., Jiang X., Xie X., Yin J., Zhang J., Liu T., Chen S., Wang Y., Zhou X., Wang Y. (2022). piR-823 inhibits cell apoptosis via modulating mitophagy by binding to PINK1 in colorectal cancer. Cell Death Dis..

[39] Tang X., Xie X., Wang X., Wang Y., Jiang X., Jiang H. (2018). The Combination of piR-823 and Eukaryotic Initiation Factor 3 B (EIF3B) Activates Hepatic Stellate Cells via Upregulating TGF-β1 in Liver Fibrogenesis. Med. Sci. Monit..

[40] Rizzo F., Rinaldi A., Marchese G., Coviello E., Sellitto A., Cordella A., Giurato G., Nassa G., Ravo M., Tarallo R. (2016). Specific patterns of PIWI-interacting small noncoding RNA expression in dysplastic liver nodules and hepatocellular carcinoma. Oncotarget.

[41] Cui L., Lou Y., Zhang X., Zhou H., Deng H., Song H., Yu X., Xiao B., Wang W., Guo J. (2011). Detection of circulating tumor cells in peripheral blood from patients with gastric cancer using piRNAs as markers. Clin. Biochem..

[42] Kasik M., Iliev R., Vychytilova P., Rybecka S., Radova L., Stanik M., Fedorko M., Dolezel J., Slaby O., Bohosova J. (2025). Circulating Serum piRNAs as Diagnostic and Prognostic Tools for Clear Cell Renal Cell Carcinoma. Cancer Genom. Proteom..

[43] Shaker F.H., Sanad E.F., Elghazaly H., Hsia S.-M., Hamdy N.M. (2024). piR-823 tale as emerging cancer-hallmark molecular marker in different cancer types: A step-toward ncRNA-precision. Naunyn-Schmiedeberg’s Arch. Pharmacol..

[44] Weng W., Liu N., Toiyama Y., Kusunoki M., Nagasaka T., Fujiwara T., Wei Q., Qin H., Lin H., Ma Y. (2018). Novel evidence for a PIWI-interacting RNA (piRNA) as an oncogenic mediator of disease progression, and a potential prognostic biomarker in colorectal cancer. Mol. Cancer.

[45] Limanówka P., Ochman B., Świętochowska E. (2023). PiRNA Obtained through Liquid Biopsy as a Possible Cancer Biomarker. Diagnostics.

[46] Cheng Y., Wang Q., Jiang W., Bian Y., Zhou Y., Gou A., Zhang W., Fu K., Shi W. (2019). Emerging roles of piRNAs in cancer: Challenges and prospects. Aging.

[47] Moyano M., Stefani G. (2015). piRNA involvement in genome stability and human cancer. J. Hematol. Oncol..

[48] Jia D.-D., Jiang H., Zhang Y.-F., Zhang Y., Qian L.-L., Zhang Y.-F. (2022). The regulatory function of piRNA/PIWI complex in cancer and other human diseases: The role of DNA methylation. Int. J. Biol. Sci..

[49] Liu Y., Dou M., Song X., Dong Y., Liu S., Liu H., Tao J., Li W., Yin X., Xu W. (2019). The emerging role of the piRNA/piwi complex in cancer. Mol. Cancer.

[50] Huang Y., Bai J.Y., Ren H.T. (2014). piRNA biogenesis and its functions. Russ. J. Bioorganic Chem..

[51] Ai L., Mu S., Sun C., Fan F., Yan H., Qin Y., Cui G., Wang Y., Guo T., Mei H. (2019). Myeloid-derived suppressor cells endow stem-like qualities to multiple myeloma cells by inducing piRNA-823 expression and DNMT3B activation. Mol. Cancer.

[52] Chen T., Mahdadi S., Vidal M., Desbène-Finck S. (2024). Non-nucleoside inhibitors of DNMT1 and DNMT3 for targeted cancer therapy. Pharmacol. Res..

[53] Yao D., Dai C., Peng S. (2011). Mechanism of the Mesenchymal–Epithelial Transition and Its Relationship with Metastatic Tumor Formation. Mol. Cancer Res..

[54] Blassl C., Kuhlmann J.D., Webers A., Wimberger P., Fehm T., Neubauer H. (2016). Gene expression profiling of single circulating tumor cells in ovarian cancer—Establishment of a multi-marker gene panel. Mol. Oncol..

[55] Tan J., Zheng B., Zhou S. (2025). Deciphering the ‘Rosetta Stone’ of ovarian cancer stem cells: Opportunities and challenges. Biochim. Biophys. Acta (BBA)—Rev. Cancer.

[56] Sohn E.J. (2022). PIK3R3, a regulatory subunit of PI3K, modulates ovarian cancer stem cells and ovarian cancer development and progression by integrative analysis. BMC Cancer.

[57] E H., Zhang L., Yang Z., Xu L., Wang T., Guo J., Xia L., Yu J., Wang H., She Y. (2024). SNAI1 promotes epithelial-mesenchymal transition and maintains cancer stem cell-like properties in thymic epithelial tumors through the PIK3R2/p-EphA2 Axis. J. Exp. Clin. Cancer Res..

[58] So J.Y., Yang H.H., Park W.Y., Skrypek N., Ishii H., Chen J.M., Lee M.P., Yang L. (2022). DNA Methyltransferase 3B–Mediated Intratumoral Heterogeneity and Therapeutic Targeting in Breast Cancer Recurrence and Metastasis. Mol. Cancer Res..

[59] Nguyen V.H.L., Hough R., Bernaudo S., Peng C. (2019). Wnt/β-Catenin Signalling in Ovarian Cancer: Insights into Its Hyperactivation and Function in Tumorigenesis. J. Ovarian Res..

[60] Li S., Han Z., Zhao N., Zhu B., Zhang Q., Yang X., Sheng D., Hou J., Guo S., Wei L. (2018). Inhibition of DNMT suppresses the stemness of colorectal cancer cells through down-regulating Wnt signaling pathway. Cell Signal..

[61] Mohapatra P., Madhulika S., Behera S., Singh P., Sa P., Prasad P., Swain R.K., Sahoo S.K. (2023). Nimbolide-based nanomedicine inhibits breast cancer stem-like cells by epigenetic reprogramming of DNMTs-SFRP1-Wnt/β-catenin signaling axis. Mol. Ther. Nucleic Acids.

[62] Liu R., Zhang Y., Ding Y., Zhang S., Pan L. (2020). Characteristics of TGFBR1–EGFR–CTNNB1–CDH1 Signaling Axis in Wnt-Regulated Invasion and Migration in Lung Cancer. Cell Transplant..

[63] Ma F., Li W., Liu C., Li W., Yu H., Lei B., Ren Y., Li Z., Pang D., Qian C. (2017). MiR-23a promotes TGF-β1-induced EMT and tumor metastasis in breast cancer cells by directly targeting CDH1 and activating Wnt/β-catenin signaling. Oncotarget.

[64] Samaei N.M., Yazdani Y., Alizadeh-Navaei R., Azadeh H., Farazmandfar T. (2014). Promoter methylation analysis of WNT/β-catenin pathway regulators and its association with expression of DNMT1 enzyme in colorectal cancer. J. Biomed. Sci..

[65] Lin J., Zeng C., Zhang J.K., Song Z., Qi N., Liu X., Zhang Z., Li A., Chen F. (2021). EFNA4 promotes cell proliferation and tumor metastasis in hepatocellular carcinoma through a PIK3R2/GSK3β/β-catenin positive feedback loop. Mol. Ther. Nucleic Acids.

[66] Yuan C., Dong H., Wu C., Liu J., Wang Z., Wang X., Ren H., Wang Z., Lu Q. (2025). EPG-5 regulates TGFB/TGF-β and WNT signalling by modulating retrograde endocytic trafficking. Autophagy.

[67] Sohn E.J., Oh S.-O. (2023). P-Element-Induced Wimpy Testis Proteins and P-Element-Induced Wimpy Testis-Interacting RNAs Expression in Ovarian Cancer Stem Cells. Genet. Test. Mol. Biomark..

[68] Wang N., Tan H.-Y., Lu Y., Chan Y.-T., Wang D., Guo W., Xu Y., Zhang C., Chen F., Tang G. (2021). PIWIL1 governs the crosstalk of cancer cell metabolism and immunosuppressive microenvironment in hepatocellular carcinoma. Signal Transduct. Target. Ther..

[69] Liu S., Yan Y., Cui Z., Feng H., Zhong F., Liu Z., Li Y., Ou X., Li W. (2023). Relationship between PIWIL1 gene polymorphisms and epithelial ovarian cancer susceptibility among southern Chinese woman: A three-center case–control study. BMC Cancer.

[70] Chen Z., Che Q., He X., Wang F., Wang H., Zhu M., Sun J., Wan X. (2015). Stem cell protein Piwil1 endowed endometrial cancer cells with stem-like properties via inducing epithelial-mesenchymal transition. BMC Cancer.

[71] Prat J. (2015). FIGO’s staging classification for cancer of the ovary, fallopian tube, and peritoneum: Abridged republication. J. Gynecol. Oncol..

[72] El-Mesallamy H.O., Hamdy N.M., Sallam A.A. (2013). Effect of obesity and glycemic control on serum lipocalins and insulin-like growth factor axis in type 2 diabetic patients. Acta Diabetol..

[73] El-Mesallamy H.O., Hamdy N.M., Zaghloul A.S., Sallam A.M. (2012). Serum retinol binding protein-4 and neutrophil gelatinase-associated lipocalin are interrelated in pancreatic cancer patients. Scand. J. Clin. Lab. Investig..

[74] El-Mesallamy H.O., Hamdy N.M., Zaghloul A.S., Sallam A.M. (2013). Clinical value of circulating lipocalins and insulin-like growth factor axis in pancreatic cancer diagnosis. Pancreas.

[75] El-Mesallamy H., Suwailem S., Hamdy N. (2007). Evaluation of C-reactive protein, endothelin-1, adhesion molecule(s), and lipids as inflammatory markers in type 2 diabetes mellitus patients. Mediat. Inflamm..

[76] El-Mesallamy H.O., Hamdy N.M., Mostafa D.M., Amin A.I. (2014). The serine protease granzyme B as an inflammatory marker, in relation to the insulin receptor cleavage in human obesity and type 2 diabetes mellitus. J. Interferon Cytokine Res..

[77] Lenart P., Novak J., Bienertova-Vasku J. (2018). PIWI-piRNA pathway: Setting the pace of aging by reducing DNA damage. Mech. Ageing Dev..

[78] Eissa S., Swellam M., el-Mosallamy H., Mourad M.S., Hamdy N., Kamel K., Zaglol A.S., Khafagy M.M., el-Ahmady O. (2003). Diagnostic value of urinary molecular markers in bladder cancer. Anticancer. Res..

[79] El-Sheikh N.M., Abulsoud A.I., Fawzy A., Wasfey E.F., Hamdy N.M. (2023). LncRNA NNT-AS1/hsa-miR-485-5p/HSP90 axis in-silico and clinical prospect correlated-to histologic grades-based CRC stratification: A step toward ncRNA Precision. Pathol. Res. Pract..

[80] Radwan S.M., Hamdy N.M., El-Mesallamy H.O. (2016). Beclin-1 and hypoxia-inducible factor-1α genes expression: Potential biomarkers in acute leukemia patients. Cancer Biomark..

[81] Atta H., Alzahaby N., Hamdy N.M., Emam S.H., Sonousi A., Ziko L. (2023). New trends in synthetic drugs and natural products targeting 20S proteasomes in cancers. Bioorganic Chem..

