# Supplementary material for: P-Element-Induced Wimpy Testis (PIWI)-Interacting RNA-823/PIWIL1/DNMT3B/CDH1 as Potential Axis to Drive EMT, Stemness, and Tumor Aggressiveness in Ovarian Cancer Tissue Samples: An Integrative Computational and Clinical Insights
Source: Int J Mol Sci. 2026 Jan 14;27(2):823. doi: 10.3390/ijms27020823 (PMC12841119; doi:10.3390/ijms27020823)
Supplement: Supplementary file 1 [file ijms-27-00823-s001.zip › ijms-4055688-supplementary.pdf]

Supplementary figures, figure legends and Tables

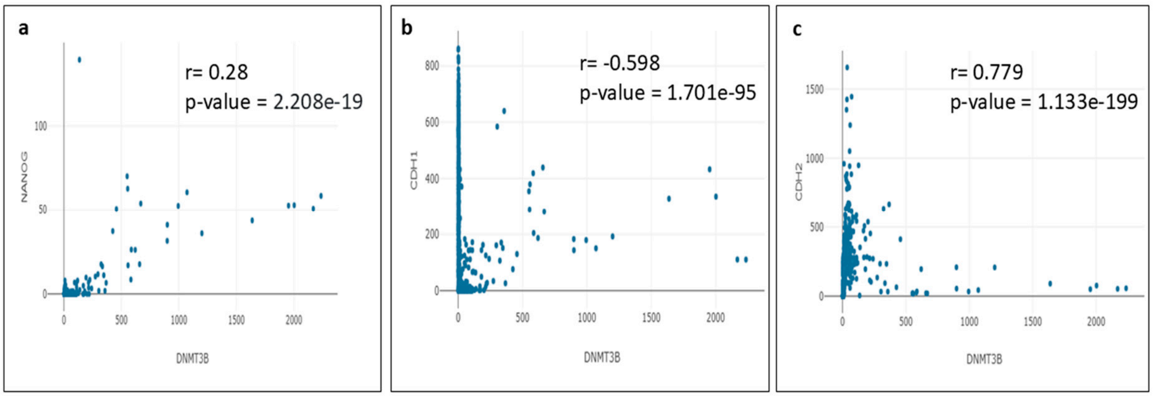

Figure S1. Spearman correlation of DNMT3B and a) NANOG b) CDH1 c) CDH2 using Organoid database

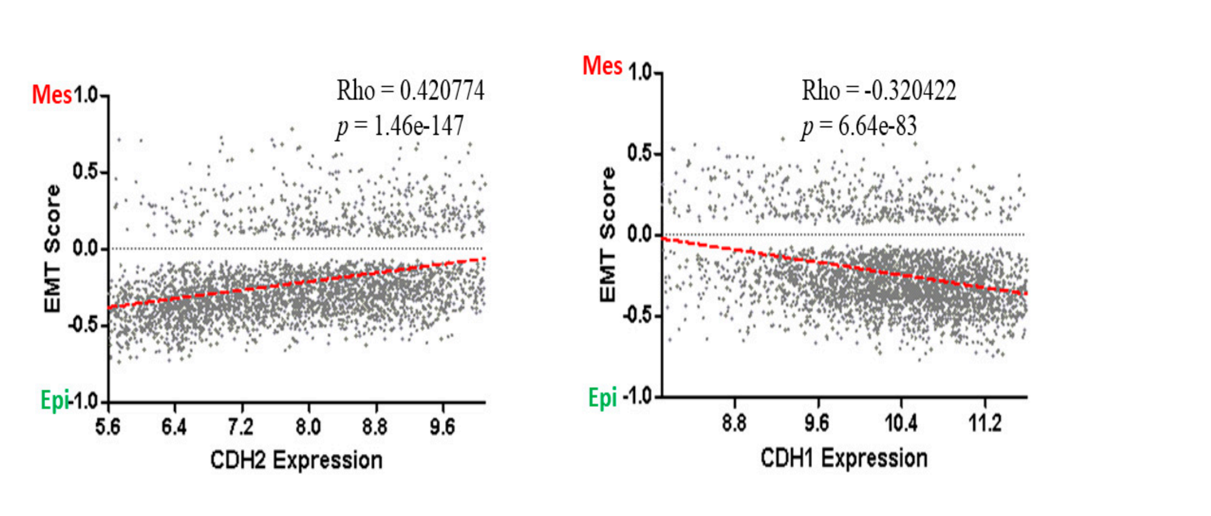

Figure S2. Correlation of CDH2 and CDH1 expression with EMT scores in ovarian cancer using CSIOVDB database. Scatter plots illustrate the relationship between EMT scores and the expression of CDH2 (N-Cadherin, left) and CDH1 (E-Cadherin, right) in ovarian cancer samples. A significant positive correlation was observed between CDH2 expression and EMT score (r= 0.4208, p = 1.46 × 10^{-147}), indicating a mesenchymal phenotype. Conversely, CDH1 expression showed a negative correlation with EMT score (r = –0.3204, p = 6.64 × 10^{-83}), reflecting its role in maintaining epithelial characteristics.

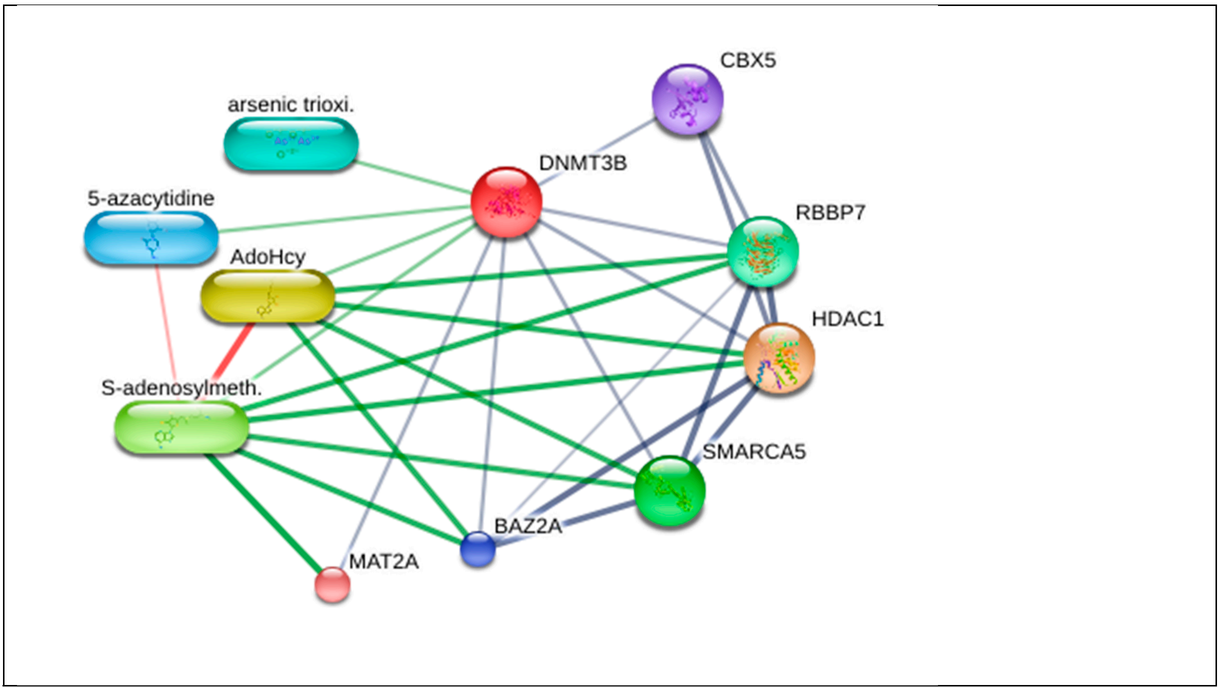

Figure S3. PPI network of DNMT3B interactions via STITCH http://stitch.embl.de/cgi/network.pl?taskId=LMs4CQ3bCuKW&sessionId=pUgfeXI7VuVI&bottom_page_content=settings

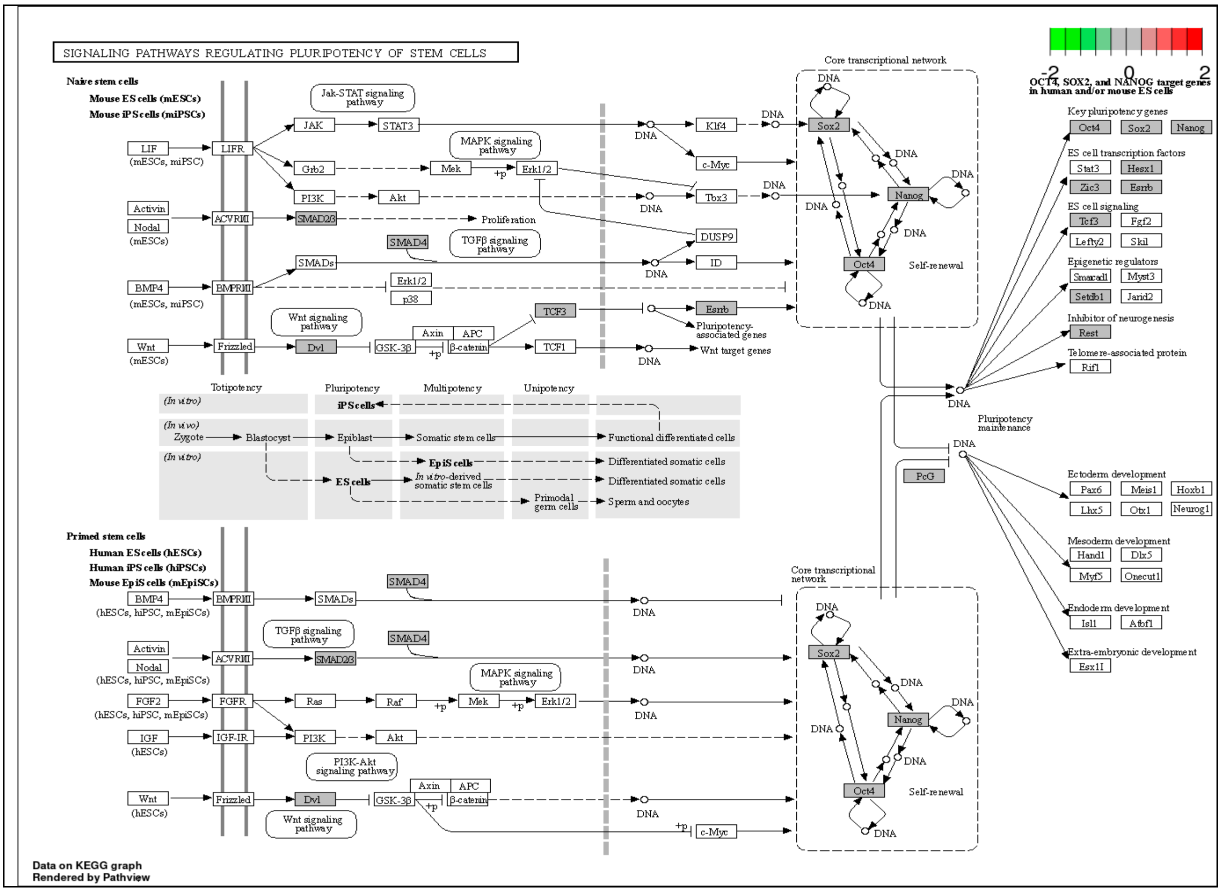

Figure S4. KEGG pathway map of the Signaling pathway regulating pluripotency of stem cells

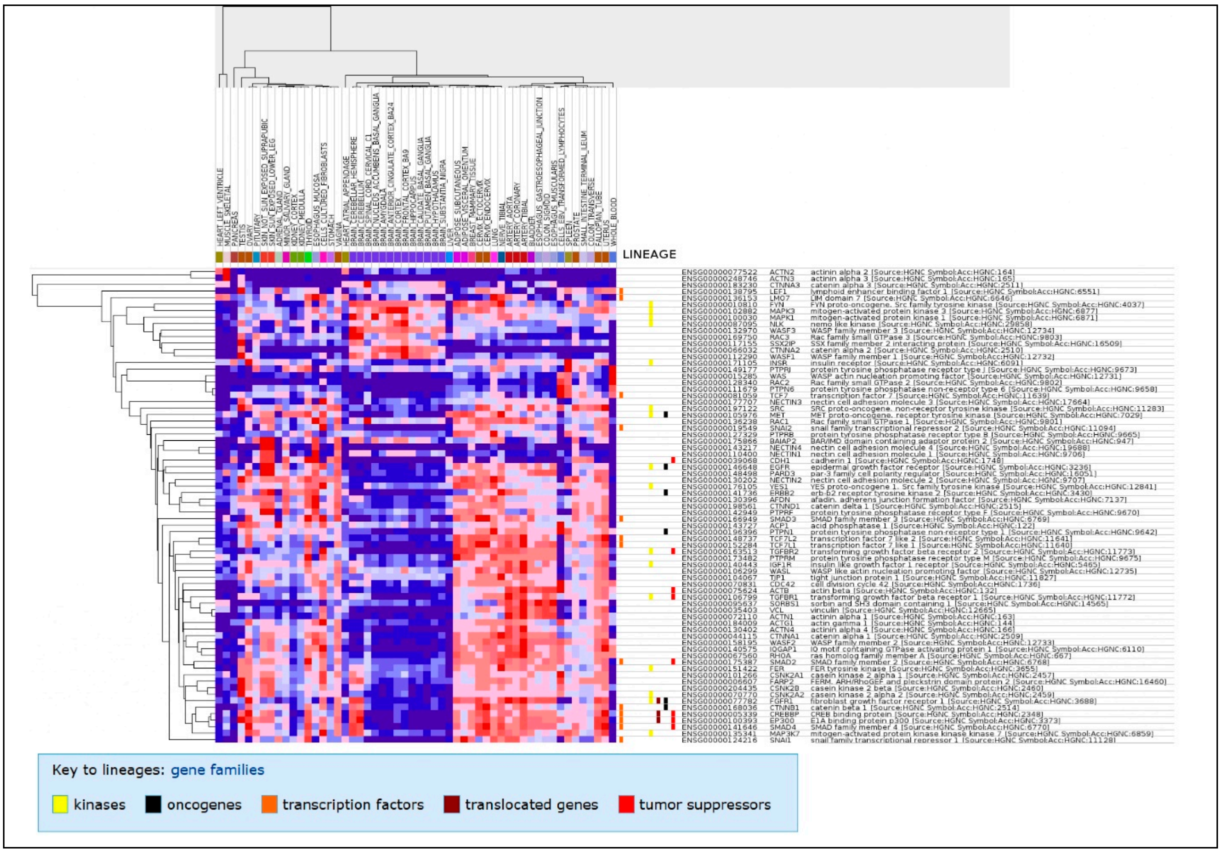

Figure S5. Heatmap of lineage-specific gene expression for KEGG Adherens Junction pathway across cancer cell lines. This heatmap presents 73 NCBI genes involved in such pathway including CDH1 (E-CAD) which is downregulated. CDH2 (N-CAD) and CTNNB1 (β-catenin) which is upregulated in OC cell lines. Gene expression levels are represented by a color gradient, where red indicates higher expression and blue indicates lower expression

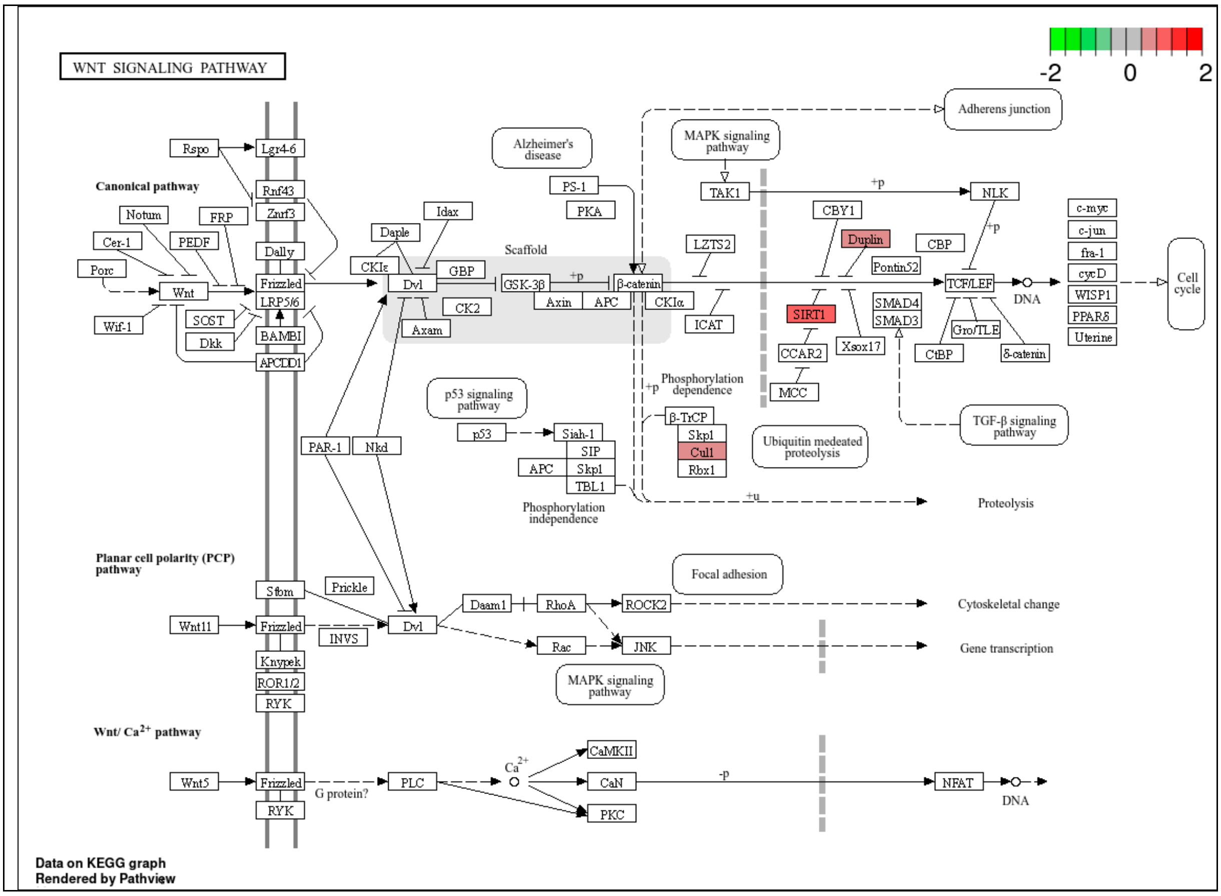

Figure S6. KEGG pathway map of the Wnt signaling pathway. Wnt signaling affects cell adhesion properties (Adherens Junction), contributing to processes like EMT

Table S1. Predicted piR-823 target genes using the sRNAtool database.

initial_alias	Converted alias	name	description
ENSG00000006194	ZNF263	ZNF263	zinc finger protein 263 [Source:HGNC Symbol;Acc:HGNC:13056]
ENSG00000013375	PGM3	PGM3	phosphoglucomutase 3 [Source:HGNC Symbol;Acc:HGNC:8907]
ENSG00000031823	RANBP3	RANBP3	RAN binding protein 3 [Source:HGNC Symbol;Acc:HGNC:9850]
ENSG00000035862	TIMP2	TIMP2	TIMP metallopeptidase inhibitor 2 [Source:HGNC Symbol;Acc:HGNC:11821]
ENSG00000043514	TRIT1	TRIT1	tRNA isopentenyltransferase 1 [Source:HGNC Symbol;Acc:HGNC:20286]
ENSG00000050820	BCAR1	BCAR1	BCAR1 scaffold protein, Cas family member [Source:HGNC Symbol;Acc:HGNC:971]
ENSG00000056998	GYG2	GYG2	glycogenin 2 [Source:HGNC Symbol;Acc:HGNC:4700]
ENSG00000063169	BICRA	BICRA	BRD4 interacting chromatin remodeling complex associated protein [Source:HGNC Symbol;Acc:HGNC:4332]
ENSG00000084073	ZMPSTE24	ZMPSTE24	zinc metallopeptidase STE24 [Source:HGNC Symbol;Acc:HGNC:12877]
ENSG00000085644	ZNF213	ZNF213	zinc finger protein 213 [Source:HGNC Symbol;Acc:HGNC:13005]
ENSG00000090971	NAT14	NAT14	N-acetyltransferase 14 (putative) [Source:HGNC Symbol;Acc:HGNC:28918]
ENSG00000092096	SLC22A17	SLC22A17	solute carrier family 22 member 17 [Source:HGNC Symbol;Acc:HGNC:23095]
ENSG00000092969	TGFB2	TGFB2	transforming growth factor beta 2 [Source:HGNC Symbol;Acc:HGNC:11768]
ENSG00000102003	SYP	SYP	synaptophysin [Source:HGNC Symbol;Acc:HGNC:11506]
ENSG00000103067	ESRP2	ESRP2	epithelial splicing regulatory protein 2 [Source:HGNC Symbol;Acc:HGNC:26152]
ENSG00000103148	NPRL3	NPRL3	NPR3 like, GATOR1 complex subunit [Source:HGNC Symbol;Acc:HGNC:14124]
ENSG00000104059	ENTREP2	ENTREP2	endosomal transmembrane epsin interactor 2 [Source:HGNC Symbol;Acc:HGNC:29075]
ENSG00000105647	PIK3R2	PIK3R2	phosphoinositide-3-kinase regulatory subunit 2 [Source:HGNC Symbol;Acc:HGNC:8980]
ENSG00000105737	GRIK5	GRIK5	glutamate ionotropic receptor kainate type subunit 5 [Source:HGNC Symbol;Acc:HGNC:4583]
ENSG00000111249	CUX2	CUX2	cut like homeobox 2 [Source:HGNC Symbol;Acc:HGNC:19347]
ENSG00000111684	LPCAT3	LPCAT3	lysophosphatidylcholine acyltransferase 3 [Source:HGNC Symbol;Acc:HGNC:30244]

ENSG00000113205	PCDHB3	PCDHB3	protocadherin beta 3 [Source:HGNC Symbol;Acc:HGNC:8688]
ENSG00000114388	NPRL2	NPRL2	NPR2 like, GATOR1 complex subunit [Source:HGNC Symbol;Acc:HGNC:24969]
ENSG00000114859	CLCN2	CLCN2	chloride voltage-gated channel 2 [Source:HGNC Symbol;Acc:HGNC:2020]
ENSG00000114999	TTL	TTL	tubulin tyrosine ligase [Source:HGNC Symbol;Acc:HGNC:21586]
ENSG00000115756	HPCAL1	HPCAL1	hippocalcin like 1 [Source:HGNC Symbol;Acc:HGNC:5145]
ENSG00000115850	LCT	LCT	lactase [Source:HGNC Symbol;Acc:HGNC:6530]
ENSG00000116031	CD207	CD207	CD207 molecule [Source:HGNC Symbol;Acc:HGNC:17935]
ENSG00000124839	RAB17	RAB17	RAB17, member RAS oncogene family [Source:HGNC Symbol;Acc:HGNC:16523]
ENSG00000125744	RTN2	RTN2	reticulon 2 [Source:HGNC Symbol;Acc:HGNC:10468]
ENSG00000125810	CD93	CD93	CD93 molecule [Source:HGNC Symbol;Acc:HGNC:15855]
ENSG00000125952	MAX	MAX	MYC associated factor X [Source:HGNC Symbol;Acc:HGNC:6913]
ENSG00000126216	TUBGCP3	TUBGCP3	tubulin gamma complex component 3 [Source:HGNC Symbol;Acc:HGNC:18598]
ENSG00000126458	RRAS	RRAS	RAS related [Source:HGNC Symbol;Acc:HGNC:10447]
ENSG00000126705	AHDC1	AHDC1	AT-hook DNA binding motif containing 1 [Source:HGNC Symbol;Acc:HGNC:25230]
ENSG00000127220	ABHD8	ABHD8	abhydrolase domain containing 8 [Source:HGNC Symbol;Acc:HGNC:23759]
ENSG00000128159	TUBGCP6	TUBGCP6	tubulin gamma complex component 6 [Source:HGNC Symbol;Acc:HGNC:18127]
ENSG00000128165	ADM2	ADM2	adrenomedullin 2 [Source:HGNC Symbol;Acc:HGNC:28898]
ENSG00000128567	PODXL	PODXL	podocalyxin like [Source:HGNC Symbol;Acc:HGNC:9171]
ENSG00000130052	STARD8	STARD8	StAR related lipid transfer domain containing 8 [Source:HGNC Symbol;Acc:HGNC:19161]
ENSG00000132330	SCLY	SCLY	selenocysteine lyase [Source:HGNC Symbol;Acc:HGNC:18161]
ENSG00000132570	PCBD2	PCBD2	pterin-4 alpha-carbinolamine dehydratase 2 [Source:HGNC Symbol;Acc:HGNC:24474]
ENSG00000134369	NAV1	NAV1	neuron navigator 1 [Source:HGNC Symbol;Acc:HGNC:15989]
ENSG00000134812	CBLIF	CBLIF	cobalamin binding intrinsic factor [Source:HGNC Symbol;Acc:HGNC:4268]

**ENSG00000135148**	**TRAFD1**	**TRAFD1**	**TRAF-type zinc finger domain containing 1 [Source:HGNC Symbol;Acc:HGNC:24808]**
**ENSG00000135736**	**CCDC102A**	**CCDC102A**	**coiled-coil domain containing 102A [Source:HGNC Symbol;Acc:HGNC:28097]**
**ENSG00000135862**	**LAMC1**	**LAMC1**	**laminin subunit gamma 1 [Source:HGNC Symbol;Acc:HGNC:6492]**
**ENSG00000136002**	**ARHGEF4**	**ARHGEF4**	**Rho guanine nucleotide exchange factor 4 [Source:HGNC Symbol;Acc:HGNC:684]**
**ENSG00000136731**	**UGGT1**	**UGGT1**	**UDP-glucose glycoprotein glucosyltransferase 1 [Source:HGNC Symbol;Acc:HGNC:15663]**
**ENSG00000137266**	**SLC22A23**	**SLC22A23**	**solute carrier family 22 member 23 [Source:HGNC Symbol;Acc:HGNC:21106]**
**ENSG00000138136**	**LBX1**	**LBX1**	**ladybird homeobox 1 [Source:HGNC Symbol;Acc:HGNC:16960]**
**ENSG00000140575**	**IQGAP1**	**IQGAP1**	**IQ motif containing GTPase activating protein 1 [Source:HGNC Symbol;Acc:HGNC:6110]**
**ENSG00000143324**	**XPR1**	**XPR1**	**xenotropic and polytropic retrovirus receptor 1 [Source:HGNC Symbol;Acc:HGNC:12827]**
**ENSG00000144815**	**NXPE3**	**NXPE3**	**neurexophilin and PC-esterase domain family member 3 [Source:HGNC Symbol;Acc:HGNC:28238]**
**ENSG00000145388**	**METTL14**	**METTL14**	**methyltransferase 14, N6-adenosine-methyltransferase non-catalytic subunit [Source:HGNC Symbol;Acc:HGNC:29330]**
**ENSG00000146216**	**TTBK1**	**TTBK1**	**tau tubulin kinase 1 [Source:HGNC Symbol;Acc:HGNC:19140]**
**ENSG00000146826**	**TRAPPC14**	**TRAPPC14**	**trafficking protein particle complex subunit 14 [Source:HGNC Symbol;Acc:HGNC:25604]**
**ENSG00000147100**	**SLC16A2**	**SLC16A2**	**solute carrier family 16 member 2 [Source:HGNC Symbol;Acc:HGNC:10923]**
**ENSG00000148343**	**MIGA2**	**MIGA2**	**mitoguardin 2 [Source:HGNC Symbol;Acc:HGNC:23621]**
**ENSG00000150051**	**MKX**	**MKX**	**mohawk homeobox [Source:HGNC Symbol;Acc:HGNC:23729]**
**ENSG00000150967**	**ABCB9**	**ABCB9**	**ATP binding cassette subfamily B member 9 [Source:HGNC Symbol;Acc:HGNC:50]**
**ENSG00000151150**	**ANK3**	**ANK3**	**ankyrin 3 [Source:HGNC Symbol;Acc:HGNC:494]**
**ENSG00000151702**	**FLI1**	**FLI1**	**Fli-1 proto-oncogene, ETS transcription factor [Source:HGNC Symbol;Acc:HGNC:3749]**
**ENSG00000153404**	**PLEKHG4B**	**PLEKHG4B**	**pleckstrin homology and RhoGEF domain containing G4B [Source:HGNC Symbol;Acc:HGNC:29399]**
**ENSG00000159216**	**RUNX1**	**RUNX1**	**RUNX family transcription factor 1 [Source:HGNC Symbol;Acc:HGNC:10471]**
**ENSG00000159259**	**CHAF1B**	**CHAF1B**	**chromatin assembly factor 1 subunit B [Source:HGNC Symbol;Acc:HGNC:1911]**
**ENSG00000162706**	**CADM3**	**CADM3**	**cell adhesion molecule 3 [Source:HGNC Symbol;Acc:HGNC:17601]**

ENSG00000163586	FABP1	FABP1	fatty acid binding protein 1 [Source:HGNC Symbol;Acc:HGNC:3555]
ENSG00000166341	DCHS1	DCHS1	dachsous cadherin-related 1 [Source:HGNC Symbol;Acc:HGNC:13681]
ENSG00000168890	TMEM150A	TMEM150A	transmembrane protein 150A [Source:HGNC Symbol;Acc:HGNC:24677]
ENSG00000169919	GUSB	GUSB	glucuronidase beta [Source:HGNC Symbol;Acc:HGNC:4696]
ENSG00000169991	IFFO2	IFFO2	intermediate filament family orphan 2 [Source:HGNC Symbol;Acc:HGNC:27006]
ENSG00000171435	KSR2	KSR2	kinase suppressor of ras 2 [Source:HGNC Symbol;Acc:HGNC:18610]
ENSG00000171786	NHLH1	NHLH1	nescient helix-loop-helix 1 [Source:HGNC Symbol;Acc:HGNC:7817]
ENSG00000172586	CHCHD1	CHCHD1	coiled-coil-helix-coiled-coil-helix domain containing 1 [Source:HGNC Symbol;Acc:HGNC:23518]
ENSG00000173465	ZNRD2	ZNRD2	zinc ribbon domain containing 2 [Source:HGNC Symbol;Acc:HGNC:11328]
ENSG00000176619	LMNB2	LMNB2	lamin B2 [Source:HGNC Symbol;Acc:HGNC:6638]
ENSG00000178175	ZNF366	ZNF366	zinc finger protein 366 [Source:HGNC Symbol;Acc:HGNC:18316]
ENSG00000178685	PARP10	PARP10	poly(ADP-ribose) polymerase family member 10 [Source:HGNC Symbol;Acc:HGNC:25895]
ENSG00000180974	OR52E4	OR52E4	olfactory receptor family 52 subfamily E member 4 [Source:HGNC Symbol;Acc:HGNC:15213]
ENSG00000181418	DDN	DDN	dendrin [Source:HGNC Symbol;Acc:HGNC:24458]
ENSG00000182256	GABRG3	GABRG3	gamma-aminobutyric acid type A receptor subunit gamma3 [Source:HGNC Symbol;Acc:HGNC:4088]
ENSG00000182612	TSPAN10	TSPAN10	tetraspanin 10 [Source:HGNC Symbol;Acc:HGNC:29942]
ENSG00000183318	SPDYE4	SPDYE4	speedy/RINGO cell cycle regulator family member E4 [Source:HGNC Symbol;Acc:HGNC:35463]
ENSG00000183496	MEX3B	MEX3B	mex-3 RNA binding family member B [Source:HGNC Symbol;Acc:HGNC:25297]
ENSG00000184182	UBE2F	UBE2F	ubiquitin conjugating enzyme E2 F (putative) [Source:HGNC Symbol;Acc:HGNC:12480]
ENSG00000184574	LPAR5	LPAR5	lysophosphatidic acid receptor 5 [Source:HGNC Symbol;Acc:HGNC:13307]
ENSG00000187140	FOXD3	FOXD3	forkhead box D3 [Source:HGNC Symbol;Acc:HGNC:3804]
ENSG00000187553	CYP26C1	CYP26C1	cytochrome P450 family 26 subfamily C member 1 [Source:HGNC Symbol;Acc:HGNC:20577]
ENSG00000187715	KBTBD12	KBTBD12	kelch repeat and BTB domain containing 12 [Source:HGNC Symbol;Acc:HGNC:25731]

**ENSG00000187922**	**LCN10**	**LCN10**	**lipocalin 10 [Source:HGNC Symbol;Acc:HGNC:20892]**
**ENSG00000188011**	**RTP5**	**RTP5**	**receptor transporter protein 5 (putative) [Source:HGNC Symbol;Acc:HGNC:26585]**
**ENSG00000188089**	**PLA2G4E**	**PLA2G4E**	**phospholipase A2 group IVE [Source:HGNC Symbol;Acc:HGNC:24791]**
**ENSG00000188807**	**TMEM201**	**TMEM201**	**transmembrane protein 201 [Source:HGNC Symbol;Acc:HGNC:33719]**
**ENSG00000196428**	**TSC22D2**	**TSC22D2**	**TSC22 domain family member 2 [Source:HGNC Symbol;Acc:HGNC:29095]**
**ENSG00000196652**	**ZKSCAN5**	**ZKSCAN5**	**zinc finger with KRAB and SCAN domains 5 [Source:HGNC Symbol;Acc:HGNC:12867]**
**ENSG00000197037**	**ZSCAN25**	**ZSCAN25**	**zinc finger and SCAN domain containing 25 [Source:HGNC Symbol;Acc:HGNC:21961]**
**ENSG00000197879**	**MYO1C**	**MYO1C**	**myosin IC [Source:HGNC Symbol;Acc:HGNC:7597]**
**ENSG00000197971**	**MBP**	**MBP**	**myelin basic protein [Source:HGNC Symbol;Acc:HGNC:6925]**
**ENSG00000204564**	**C6orf136**	**C6orf136**	**chromosome 6 open reading frame 136 [Source:HGNC Symbol;Acc:HGNC:21301]**
**ENSG00000205755**	**CRLF2**	**CRLF2**	**cytokine receptor like factor 2 [Source:HGNC Symbol;Acc:HGNC:14281]**
**ENSG00000205755**	**CRLF2**	**CRLF2**	**cytokine receptor like factor 2 [Source:HGNC Symbol;Acc:HGNC:14281]**
**ENSG00000206077**	**ZDHHC11B**	**ZDHHC11B**	**zinc finger DHHC-type containing 11B [Source:HGNC Symbol;Acc:HGNC:32962]**
**ENSG00000224982**	**TMEM233**	**TMEM233**	**transmembrane protein 233 [Source:HGNC Symbol;Acc:HGNC:37219]**
**ENSG00000235173**	**HGH1**	**HGH1**	**HGH1 homolog [Source:HGNC Symbol;Acc:HGNC:24161]**
**ENSG00000254245**	**PCDHGA3**	**PCDHGA3**	**protocadherin gamma subfamily A, 3 [Source:HGNC Symbol;Acc:HGNC:8701]**
**ENSG00000264324**	**None**	**None**	**None**
**ENSG00000264364**	**DYNLL2**	**DYNLL2**	**dynein light chain LC8-type 2 [Source:HGNC Symbol;Acc:HGNC:24596]**
**ENSG00000267680**	**ZNF224**	**ZNF224**	**zinc finger protein 224 [Source:HGNC Symbol;Acc:HGNC:13017]**
**ENSG00000273820**	**USP27X**	**USP27X**	**ubiquitin specific peptidase 27 X-linked [Source:HGNC Symbol;Acc:HGNC:13486]**
**ENSG00000277150**	**F8A3**	**F8A3**	**coagulation factor VIII associated 3 [Source:HGNC Symbol;Acc:HGNC:31850]**
**ENSG00000277932**	**OR52E5**	**OR52E5**	**olfactory receptor family 52 subfamily E member 5 [Source:HGNC Symbol;Acc:HGNC:15214]**
**ENSG00000283247**	**CCDC201**	**CCDC201**	**coiled-coil domain containing 201 [Source:HGNC Symbol;Acc:HGNC:54081]**

**ENSG00000284989**	**None**	**None**	**None**
